# STAT1 gain-of-function heterozygous cell models reveal diverse interferon-signature gene transcriptional responses

**DOI:** 10.1038/s41525-021-00196-7

**Published:** 2021-05-14

**Authors:** Ori Scott, Kyle Lindsay, Steven Erwood, Antonio Mollica, Chaim M. Roifman, Ronald D. Cohn, Evgueni A. Ivakine

**Affiliations:** 1 Genetics and Genome Biology Program, The Hospital for Sick Children Research Institute, Toronto, ON Canada; 2 Division of Clinical Immunology and Allergy, Hospital for Sick Children, University of Toronto, Toronto, ON Canada; 3 Institute of Medical Science, University of Toronto, Toronto, ON Canada; 4 Department of Molecular Genetics, University of Toronto, Toronto, ON Canada; 5 Department of Biochemistry, University of Toronto, Toronto, ON Canada; 6 Canadian Center for Primary Immunodeficiency and The Jeffrey Modell Research Laboratory for the Diagnosis of Primary Immunodeficiency, The Hospital for Sick Children and The University of Toronto, Toronto, ON Canada; 7 Division of Clinical and Metabolic Genetics, Hospital for Sick Children, University of Toronto, Toronto, ON Canada; 8 Department of Physiology, University of Toronto, Toronto, ON Canada

**Keywords:** Primary immunodeficiency disorders, Molecular medicine, Gene expression, Disease genetics

## Abstract

Signal transducer and activator of transcription 1 (STAT1) gain-of-function (GOF) is an autosomal dominant immune disorder marked by wide infectious predisposition, autoimmunity, vascular disease, and malignancy. Its molecular hallmark, elevated phospho-STAT1 (pSTAT1) following interferon (IFN) stimulation, is seen consistently in all patients and may not fully account for the broad phenotypic spectrum associated with this disorder. While over 100 mutations have been implicated in STAT1 GOF, genotype–phenotype correlation remains limited, and current overexpression models may be of limited use in gene expression studies. We generated heterozygous mutants in diploid HAP1 cells using CRISPR/Cas9 base-editing, targeting the endogenous *STAT1* gene. Our models recapitulated the molecular phenotype of elevated pSTAT1, and were used to characterize the expression of five IFN-stimulated genes under a number of conditions. At baseline, transcriptional polarization was evident among mutants compared with wild type, and this was maintained following prolonged serum starvation. This suggests a possible role for unphosphorylated STAT1 in the pathogenesis of STAT1 GOF. Following stimulation with IFNα or IFNγ, differential patterns of gene expression emerged among mutants, including both gain and loss of transcriptional function. This work highlights the importance of modeling heterozygous conditions, and in particular transcription factor-related disorders, in a manner which accurately reflects patient genotype and molecular signature. Furthermore, we propose a complex and multifactorial transcriptional profile associated with various *STAT1* mutations, adding to global efforts in establishing STAT1 GOF genotype–phenotype correlation and enhancing our understanding of disease pathogenesis.

## Introduction

Signal transducer and activator of transcription (STAT) is a family of seven structurally homologous transcription factors, activated downstream of various cytokine, growth factor, and hormone receptors. At rest, STAT molecules are found in a latent state in the cytoplasm. After receptor ligation, canonical STAT activation follows a common sequence, starting with recruitment of tyrosine kinases from the Janus-Kinase (JAK) family, which phosphorylate the cytoplasmic portion of the receptor to form a docking site for STAT. This is followed by STAT recruitment, tyrosine phosphorylation, and multimerization to form active transcription factors which then migrate to the nucleus^1–5^. Within the STAT family, STAT1 is pivotal in mediating transcriptional responses to cytokines of the interferon (IFN) family, as well as interleukin-27 (IL-27). This is achieved by the formation of transcription complexes, known as interferon-stimulated gene factor 3 (ISGF3) and gamma-activating factor (GAF). ISGF3 is a hetero-trimer consisting of STAT1, STAT2, and IFN-regulatory factor 9 (IRF9). It is primarily formed in the context of type I and III IFN stimulation, and binds to interferon-stimulated response element to regulate gene expression. In contrast, GAF is a STAT1 homodimer, predominantly activated in response to type II IFN and IL-27, which exerts its transcriptional activity by binding to gamma-activating sequence within gene promoters^2–6^.

Monogenic defects in the *STAT1* gene have been implicated in three distinct human disorders to date. Autosomal recessive complete loss of function (LOF) leads to severe and early-onset susceptibility to viral and mycobacterial infections. Individuals harboring two hypomorphic alleles display a milder form of this disease^7–10^. A second entity, caused by heterozygous dominant-negative mutations, is characterized by Mendelian susceptibility to mycobacterial disease^11,12^. The third disorder, STAT1 gain-of-function (GOF), was first described among a subset of individuals with chronic mucocutaneous Candidiasis and autoimmune thyroid disease, harboring heterozygous point mutations in *STAT1*^13,14^. The molecular hallmark of the disease was defined as increased levels of phosphorylated STAT1 (with respect to the Tyrosine-701 residue) in response to IFN stimulation^14^. Since its first description in 2011, STAT1 GOF has been diagnosed in hundreds of patients, and its phenotypic spectrum expanded^15,16^. Infectious predisposition includes fungal, bacterial, viral, opportunistic, and mycobacterial infections. Over one-third of patients display autoimmune features, with hypothyroidism, type 1 diabetes, and cytopenias being common manifestations. Vascular abnormalities, notably intracranial and aortic aneurysms, have been described at an increased frequency compared with the general population. Malignancies, in particular squamous cell carcinoma, are seen in up to 5% of patients^15–20^.

In the decade since STAT1 GOF was first described, strides have been made in characterizing the disorder and its underlying pathophysiology. A prominent example is the impaired Th17 response observed in most patients, which has been linked to predisposition to fungal and bacterial infections^14,20,21^. From an autoimmune standpoint, impaired type I IFN response has been proposed as a possible contributory mechanism, given the heightened IFN signature associated with other autoimmune and inflammatory conditions^22–24^. Indeed, alterations in IFN-related gene expression have been found in some patients with STAT1 GOF and clinical features of autoimmunity^25^. However, many underlying disease mechanisms have remained elusive, and the genotype–phenotype correlation among patients remains poorly defined. Recent works reported that the presence of a severe complication, such as invasive infection, cancer, symptomatic aneurysm, and in some cases severe autoimmunity, substantially worsened the prognosis and lowered survival^15,26^. Unfortunately, our ability to predict which patients would be more prone to developing such complications based on their specific mutations is greatly limited. Therefore, the need for developing tools to study the variability across *STAT1* GOF mutations is dire.

In studying the differences across *STAT1* GOF mutations, the use of cell models offers a well-controlled, accessible and non-invasive tool. Previous studies utilizing overexpression models generated in *STAT1*-null U3 fibrosarcoma cells (and more recently, HEK293 cells) have been instrumental in elucidating differences among mutations with respect to STAT1 phosphorylation kinetics, nuclear migration, and accumulation^14,27–31^. However, such models involve the expression of *STAT1* under an exogenous promoter, and do not capture the heterozygous nature of the condition. In the context of a delicately regulated transcription factor, overexpression models may therefore be limited in their portrayal of gene expression patterns downstream of STAT1. The current study describes our use of the Clustered Regularly Interspaced Short Palindromic Repeats (CRISPR)/Cas9 system, and in particular CRISPR/Cas9 base-editing, to generate a series of heterozygous cell models harboring known GOF point mutations, within the endogenous *STAT1* gene. We further use these models to show that *STAT1* GOF mutations result in different patterns of interferon-stimulated gene (ISG) expression, both at baseline and following stimulation with IFNα or IFNγ. We propose that such models may enhance our understanding of this intricate immune disorder, as well as genotype–phenotype correlation among various *STAT1* GOF mutations.

## Results

### Diploid HAP1 cells were chosen for heterozygous mutation modeling

For the purpose of our model generation we have used HAP1, a cell line originally derived from the KBM-7 chronic myelogenous leukemia cell line. HAP1 were previously used to study cellular responses to IFN types I, II, and III, and have well-characterized transcriptional responses to IFN type I and II^32–34^. In addition, they are readily amenable to transfection and CRISPR/Cas genome-editing^35^. Although HAP1 cells are originally near-haploid, like other haploid cell lines they are known to spontaneously diploidize in cell culture over time^36^. HAP1 cells used in this study underwent cell cycle analysis, comparing their DNA content with that of known diploid cells [wild-type (WT) human fibroblasts]. The mean fluorescence intensity (MFI) ratios of HAP1/fibroblasts for the G_0_/G_1_ and G_2_/M peaks were calculated to be 1.07 and 1.11, respectively, confirming our HAP1 cells to be fully diploid, and therefore suitable for heterozygous mutation modeling (Supplementary Fig. 1).

### Heterozygous *STAT1* mutants were generated using CRISPR/Cas9 base-editing

**Fig. 1:**
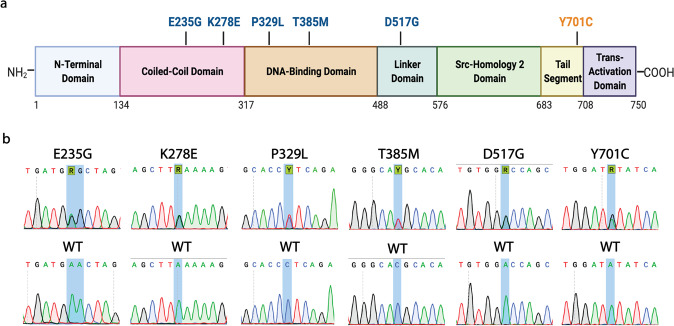
*STAT1* mutations modeled in this work. **a** Schematic representation of *STAT1* mutations selected for modeling and their respectively affected protein domains. Mutations designated in blue (E235G, K278E, P329L, T385M, D517G) are “gain-of-function” mutations, while orange (Y701C) designates a “loss-of-function” mutation. **b** Sanger sequencing confirmation of generated cell models, compared with their respective wild-type counterparts. Highlighted nucleotides denote the edited base. Note that for E235G, editing of an adjacent “A” took place on both alleles, resulting in a silent bystander mutation. This yielded the trinucleotide change: GAA/GAA → GAG/GGG, leading to the amino acid substitution: glutamine/glutamine → glutamine/glycine. This corresponds with the same amino acid change found in patients affected by the E235G mutation.

The following validated GOF transition mutations were chosen for modeling: E235G^37^, K278E^38^ (both in the coiled-coil domain), P329L^39,40^, T385M^16,41–49^ (in the DNA-binding domain), and D517G^15^ (in the linker domain). The dominant-negative mutation Y701C^50^, affecting the JAK-phosphorylated residue Y701, was chosen for comparative modeling as well. A visual representation of modeled mutations within their respective protein domains is presented in Fig. 1a. A schematic outline of the workflow for generating and verifying the *STAT1* mutants is provided as Supplementary Fig. 2. For information regarding clinical manifestations described for each mutation, please refer to Table 1.

**Table 1:** Clinical manifestations of *STAT1* mutations modeled in this work.

Mutation	Designation	Fungal infections	Bacterial infections	Viral infections	Mycobacterial infections	Autoimmunity/endocrinopathy	Vascular abnormalities	Malignancy	Other
E235G (c.704A>G)	AD GOF	CMCC	Sinopulmonary infections, bacterial skin infections with abscess formation	Recurrent HSV labialis	Mycobacterial pneumonia	Alopecia	Carotid and celiac/splenic artery dissection	HPV+squamous cell carcinoma, basal cell carcinoma	ND
K278E (c.832A>G)	AD GOF	CMCC	Bacterial skin infections	Recurrent HSV stomatitis, recurrent Herpes zoster	ND	Positive auto-antibodies (ANA, microsomal, anti-TSH, anti-IL-17F)	ND	ND	ND
P329L (c.986C>T)	AD GOF	CMCC	Yes, no details provided	Recurrent HSV stomatitis	ND	Autoimmune hemolytic anemia, red blood cell aplasia	ND	ND	ND
T385M (c.1154C>T)	AD GOF	CMCC, invasive fungal infections	Sinopulmonary infections	EBV, CMV, recurrent severe VZV	Variety of mycobacterial infections reported	Enteropathy, T1DM, thyroiditis, autoimmune cytopenias, vitiligo, growth hormone deficiency, autoimmune hepatitis	Intracranial aneurysms	ND	Recurrent fractures, eczema, chronic adenopathy, multifocal leuko-encephalopathy
D517G (c.1550A>G)	AD GOF	CMCC, invasive fungal infections	ND	ND	ND	ND	ND	ND	ND
Y701C (c.2102A>G)	AD LOF	ND	ND	ND	Invasive mycobacterial infections	ND	ND	ND	Multifocal osteomyelitis

*AD* autosomal dominant, *ANA* anti-nuclear antibody, *CMCC* chronic mucocutaneous Candidiasis, *CMV* cytomegalovirus, *EBV* Epstein-Barr virus, *GOF* gain-of-function, *HPV* human papillomavirus, *HSV*, human herpesvirus, *LOF* loss-of-function, *ND* not described, *T1DM* Type 1 diabetes mellitus, *TSH* thyroid-stimulating hormone, *VZV* Varicella zoster virus.

To generate chosen mutations, single-guide RNA (sgRNA) targeting the Cas9 base editor to the region of interest were cloned into the BPK1520_puroR plasmid. Resultant plasmids, coding for the desired sgRNA as well as a puromycin resistance cassette, were delivered by lipofection into HAP1 cells, concurrently with an additional plasmid coding the respective Cas9 base editor (SpCas9 ABEmax^51^, SpG CBE4max, or SpRY ABEmax/CBE4max)^52^. Following puromycin selection to enrich for transfected cells, base-editing efficiency was evaluated in the bulk population by sequencing. Overall, editing efficiency ranged from 15 to 77% for the desired target nucleotide. As base editors each have a characteristic “editing window”, adjacent nucleotides to the nucleotide of interest may be prone to “bystander” editing. As an example, if two adjacent adenine residues are both within the editing window for an adenine base editor, both may be targeted and converted to guanine, albeit at different frequencies depending on their position within the editing window. In this study, bystander mutations involving editing of adjacent bases within the editing window occurred at a frequency of 1–38%. Cells from the bulk population were single-cell sorted, and resultant single clones were screened by Sanger sequencing for the presence of the desired mutation in a heterozygous state, and for the absence of non-silent bystander mutations. For all mutations, the number of clones required to be screened to identify a desired clone ranged from 12 to 39. For one mutation, E235G, only clones containing a second, silent mutation in an adjacent base could be identified. However, these clones recapitulated the desired amino acid change and were therefore deemed appropriate for further downstream work. Altogether, all chosen amino acid changes could be modeled using base-editing. For each mutation generated, downstream analysis was carried out on a minimum of two independent clones. A summary of base-editing performed in this work, including sgRNA and base editors used, frequency of editing events in the bulk population, and number of clones required to screen to find a heterozygous mutation, is provided in Table 2. Sanger sequencing validation of all mutations is provided in Fig. 1b.

**Table 2:** Summary of base-editing features for *STAT1* mutation generation.

Mutation	Desired trinucleotide change	Single-guide RNA sequence^a^	Base editor used	Protospacer adjacent motif (PAM) sequence	On-target base-editing (%)	Off-target changes within the editing window (%)	Number of clones screened to obtain the desired mutation in a heterozygous state
E235G (c.704A>G)	GAA>GGA	GATGAACTAGTGGAGTGGAAG	SpCas9 ABEmax	CGG	50	38	39^b^
K278E (c.832A>G)	AAA>GAA	GCTTAAAAAGTTGGAGGAAT	SpCas9 ABEmax	TGG	67	7	17
P329L (c.986C>T)	CCT>CTT	GCACCCTCAGAGGCCGCTGGT	SpRY CBE4max	CTT	15	10	16
T385M (c.1154C>T)	ACG>ATG	GGGCACGCACACAAAAGTGA	SpG CBE4max	TGA	21	4	14
D517G (c.1550A>G)	GAC>GGC	gTGTGGACCAGCTGAACATGT	SpCas9 ABEmax	TGG	77	2	12
Y701C (c.2102A>G)	TAT>TGT	gTGGATATATCAAGACTGAGT	SpRY ABEmax	TGA	36	15	24

^a^Non-template “g” appended to single-guide RNA to facilitate transcription downstream of the U6 promoter designated in lowercase.

^b^Resultant clone contains a silent bystander nucleotide change within the editing window.

### Immunoblotting for pSTAT1 validated the molecular designation of generated *STAT1* mutants

**Fig. 2:**
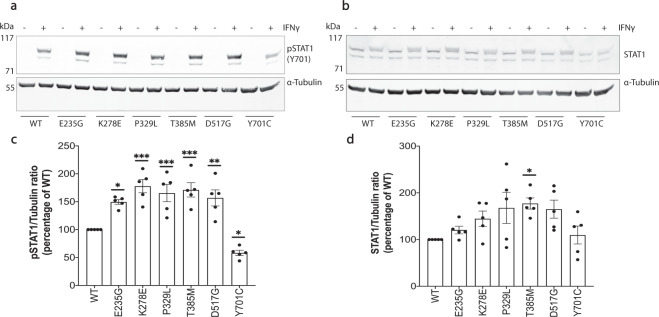
Immunoblot analysis of pSTAT1 (Tyr701) and total STAT1 levels among STAT1 mutants following IFNγ stimulation. Levels of pSTAT1 (**a**) and total STAT1 (**b**) were measured in whole-cell protein lysates at baseline and following a 60-min stimulation with IFNγ (10 ng/mL), normalized to a loading control (α-Tubulin). Densitometry analysis results from five independent experiments were plotted for pSTAT1 (**c**) and STAT1 levels (**d**) following IFNγ stimulation and compared with WT. Results are presented as mean ± standard error of mean. Statistical analysis: one-way ANOVA with Dunnett’s post hoc test (*p < 0.05, **p < 0.01, ***p < 0.001,****p < 0.0001).

In order to establish the validity of our newly generated cell models, we proceeded to validate their designation as “GOF” or “LOF” based on Tyrosine-701 phosphorylation in response to IFN. To this end, cells were stimulated with IFNγ at a dose of 10 ng/mL for a period of 60 min. pSTAT1(Y701) and total STAT1 were measured by immunoblotting at baseline in unstimulated cells, as well as following stimulation. Measurements were repeated over five independent experiments and densitometry analysis performed (Fig. 2 and Supplementary Fig. 3a, b). Detailed densitometry values are provided in Supplementary Tables 1 and 2. At baseline, pSTAT1 was not detectable in any of the samples. Following IFNγ stimulation, levels of pSTAT1 increased across all samples, but were significantly higher across all GOF mutants compared with WT [E235G (*p* < 0.05), K278E, P329L, T385M (*p* < 0.001), D517G (*p* < 0.01); one-way ANOVA with Dunnett’s post hoc test]. By the same token, pSTAT1 was lower in the Y701C LOF mutant compared with WT after stimulation (*p* < 0.05). These results establish that modeled heterozygous *STAT1* mutations in HAP1 cells lead to the same functional consequences with respect to protein phosphorylation as are seen in patients. Total STAT1 was comparable to WT among most mutants.

### Gene expression studies demonstrated baseline polarization among *STAT1* mutants

To evaluate the transcriptional impact of the various *STAT1* mutations, we used quantitative real-time PCR (qRT-PCR) to assess differences in ISG expression under a number of conditions, including baseline, serum starvation, and stimulation with IFN type I and II. A visual summary of STAT1 signaling associated with the above conditions is presented in Supplementary Fig. 4. A set of five ISG was chosen consisting of *GBP1, IFIT2, IRF1, APOL6*, and *OAS1*. These genes were selected as they are known to increase in human cells at least twofold following either IFNα or IFNγ stimulation^53^. Moreover, previous studies specifically done in HAP1 cells showed these genes to increase at least twofold following stimulation with either type I or II IFN stimulation^34^.

**Fig. 3:**
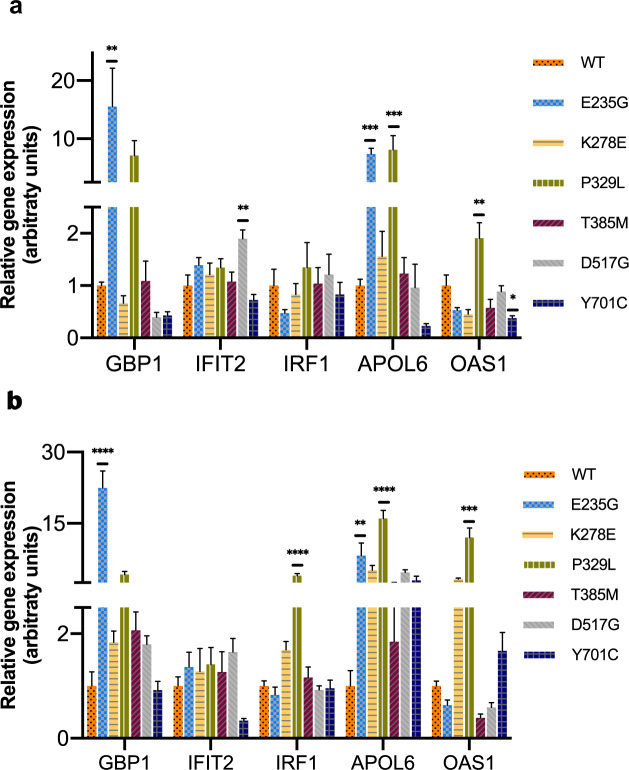
Relative gene expression of interferon-stimulated genes (ISG) at baseline and following serum starvation. **a** mRNA expression levels were measured for five ISG (*GBP1, IFIT2, IRF1, APOL6, OAS1*) at baseline in cells grown under normal serum conditions; **b** mRNA expression levels for five ISG measured in cells following 24-h of serum starvation. Expression levels were normalized to housekeeping *GAPDH* expression and plotted relatively to wild type for each experiment. Pooled data from at least five experiments are presented. Data are represented as mean ± standard error of mean. Statistical analysis: one-way ANOVA with Dunnett’s post hoc test (*p < 0.05, **p < 0.01, ***p < 0.001, ****p < 0.0001).

At baseline, significant differences in gene expression among WT and some of the mutants were already noted, involving a mixed pattern of both increased and decreased expression (Fig. 3a and Table 3). The most prominent mutants showing elevated expression (two genes each) were E235G and P329L; E235G showed increased expression of *GBP1* (*p* < 0.01) and *APOL6* (*p* < 0.001), while P329L demonstrated increased *APOL6* (*p* < 0.001) and *OAS1* expression (*p* < 0.01). Other changes noted included reduced *OAS1* expression in Y701C (*p* < 0.05), and elevated *IFIT2* expression in D517G (*p* < 0.01). No baseline differences were noted between T385M and WT, or between K278E and WT. In order to ensure that the observed transcriptional differences were inherent to the mutants, rather than a result of external cell-culture cytokine/growth factor stimuli, gene expression was measured following 24 h of serum starvation (Fig. 3b and Table 3). In the context of serum starvation, the mutants E235G and P329L maintained a profile of elevated gene expression. E235G demonstrated increased expression of *GBP1* (*p* < 0.0001) and *APOL6* (*p* < 0.01) compared to WT, while P329L showed enhanced expression of *APOL6* (*p* < 0.0001), *OAS1* (*p* < 0.001) and in addition, *IRF1* (*p* < 0.0001). Mutants that were previously no different than WT (K278E and T385M) with respect to all genes measured remained so under serum starvation. In the context of these results, and given that serum starvation in and of itself may impact STAT1 activation and gene transcription in an IFN-independent manner^54^, we elected to proceed with IFN stimulation experiments under normal cell culture conditions, as described by others ^14,16,25,27,28,30,31^.

**Table 3:** Summary of differences in relative expression or fold increase across *STAT1* mutants compared with wild type under various conditions.

Mutation	Condition/stimulation	GBP1	IFIT2	IRF1	APOL6	OAS1
E235G	Baseline (full serum)^a^	↑	NS	NS	↑	NS
	Serum starvation^b^	↑	NS	NS	↑	NS
	IFNα^c^	NS	↑	↑	NS	NS
	IFNγ^d^	↓	NS	NS	↓	↑
K278E	Baseline (full serum)^a^	NS	NS	NS	NS	NS
	Serum starvation^b^	NS	NS	NS	NS	NS
	IFNα^c^	↓	NS	NS	↓	↓
	IFNγ^d^	NS	↑	NS	NS	NS
P329L	Baseline (full serum)^a^	NS	NS	NS	↑	↑
	Serum starvation^b^	NS	NS	↑	↑	↑
	IFNα^c^	↓	NS	NS	↓	↓
	IFNγ^d^	↓	NS	NS	↓	NS
T385M	Baseline (full serum)^a^	NS	NS	NS	NS	NS
	Serum starvation^b^	NS	NS	NS	NS	NS
	IFNα^c^	↑	↑	↑	↑	↑
	IFNγ^d^	NS	NS	NS	↓	NS
D517G	Baseline (full serum)^a^	NS	↑	NS	NS	NS
	Serum starvation^b^	NS	NS	NS	NS	NS
	IFNα^c^	NS	NS	NS	↓	↓
	IFNγ^d^	NS	NS	NS	↓	NS
Y701C	Baseline (full serum)^a^	NS	NS	NS	NS	↓
	Serum starvation^b^	NS	NS	NS	NS	NS
	IFNα^c^	↓	↓	↓	↓	NS
	IFNγ^d^	↓	↓	↓	↓	NS

*NS* not significant.

^a^Relative gene expression compared with wild type under full-serum culture conditions without cytokine stimulation.

^b^Relative gene expression compared with wild type following 24 h of low-serum conditions without cytokine stimulation.

^c^Fold increase in gene expression from baseline compared with wild type following 6 h of stimulation with IFNα (10 ng/mL).

^d^Fold increase in gene expression from baseline compared with wild type following 6 h of stimulation with IFNγ (10 ng/mL).

### *STAT1* mutants displayed a differential response to IFNα stimulation involving both loss and gain of transcriptional function

**Fig. 4:**
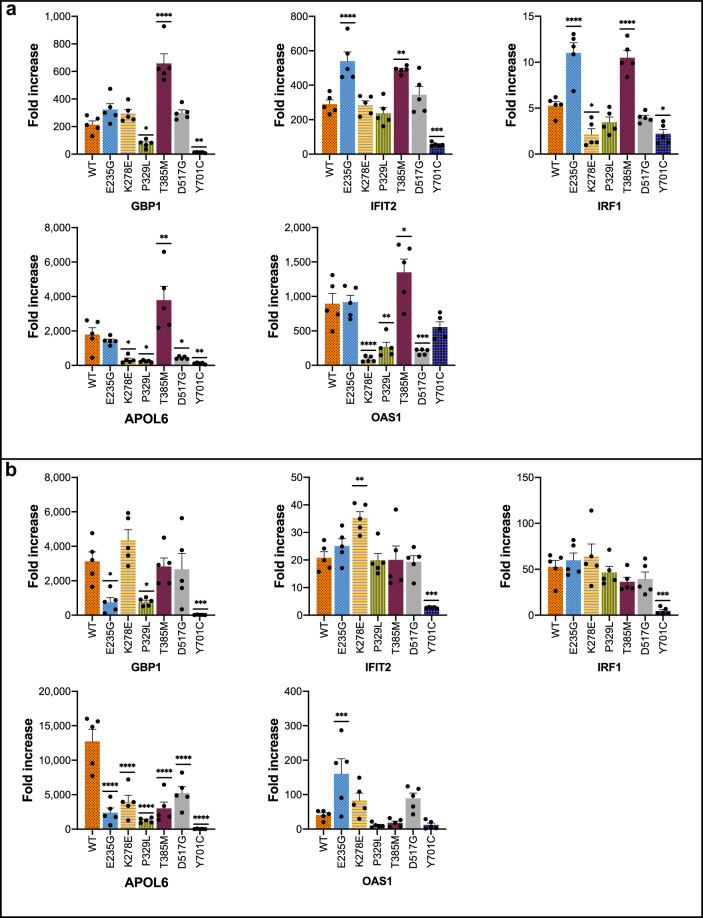
Relative gene expression of interferon-stimulated genes (ISG) following IFNα or IFNγ stimulation. mRNA expression levels were measured for five ISG (*GBP1, IFIT2, IRF1, APOL6, OAS1*) at baseline and following 6 h of stimulation with IFNα (**a**) or IFNγ (**b**). Expression levels were normalized to housekeeping *GAPDH* and plotted as fold increase from baseline. Pooled data from at least five experiments are presented. Data are represented as mean ± standard error of mean. Statistical analysis: one-way ANOVA with Dunnett’s post-hoc test (*p < 0.05, **p < 0.01, ***p < 0.001, ****p < 0.0001).

After establishing baseline expression levels, transcriptional responses (fold increase in expression after stimulation) were measured following a 6-h stimulation with IFNα (10 ng/mL) (Fig. 4a and Table 3). Of all GOF mutants, only T385M showed an elevated fold change (FC) across all genes measured compared to WT [*GBP1* (*p* < 0.0001), *IFIT2* (*p* < 0.01), *IRF1* (*p* < 0.0001), *APOL6* (*p* < 0.01), *OAS1* (*p* < 0.05)]. E235G, which had an elevated baseline expression of *GBP1* and *APOL6*, showed an elevated FC in the expression of *IFIT2* (*p* < 0.0001) and *IRF1* (*p* < 0.0001), with no difference in FC with respect to other genes. In contrast, P329L, which previously showed increased baseline expression of *APOL6* and *OAS1*, showed a reduced FC in the expression of *GBP1* (*p* < 0.05), *APOL6* (*p* < 0.05), and *OAS1* (*p* < 0.01) compared with WT. Decreased FC compared to WT was also seen in K278E [*IRF1* (*p* < 0.05), *APOL6* (*p* < 0.05), *OAS1* (*p* < 0.0001)], and D517G [*APOL6* (*p* < 0.05), *OAS1* (*p* < 0.001)]. The LOF mutant Y701C was marked by reduced FC across all but one gene compared to WT [*GBP1* (*p* < 0.01), *IFIT2* (*p* < 0.001), *IRF1* (*p* < 0.05), *APOL6* (*p* < 0.01)].

### Transcriptional response of *STAT1* mutants to IFNγ stimulation differed from IFNα responses

We sought to determine whether transcriptional responses of *STAT1* mutants to IFNa could accurately predict their responses to stimulation with IFNγ (Fig. 4b and Table 3). Following a 6-h stimulation with IFNγ (10 ng/mL), three mutants showed vastly different transcriptional responses compared with those seen following IFNα stimulation. T385M, which previously showed a transcriptional GOF with respect to all genes following IFNα stimulation, now showed a reduced FC of *APOL6* (*p* < *0.0001*), with no other differences compared with WT. E235G previously demonstrated elevated FC in expression of *IFIT2* and *IRF1* in response to IFNa, whereas no differences from WT were seen in these genes with IFNγ stimulation. In contrast, FC of *GBP1* (*p* < 0.05) and *APOL6* (*p* < 0.0001) were now decreased in E235G, and that of *OAS1* increased (*p* < 0.001) compared to WT. One more mutant showing substantial differences in responses to IFNγ and IFNα was K278E, while IFNα stimulation resulted in reduced FC of *GBP1*, *APOL6*, and *OAS1* compared with WT, IFNγ stimulation caused an increased FC in *IFIT2* (*p* < 0.01), with no significant differences with respect to other genes. Mutants showing more similar trends in response to both IFNγ and IFNα included P329L, D517G, and the LOF mutant Y701C. P329L again showed reduced FC of *GBP1* (*p* < 0.05) and *APOL6* (*p* < 0.0001), though FC for *OAS1* was no different than WT. D517G demonstrated reduced FC in *APOL6* (*p* < 0.0001), but no difference in FC of *OAS1*. Y701C showed consistent responses to IFNγ and IFNα, with reduced FC seen again for *GBP1* (*p* < 0.001), *IFIT2* (*p* < 0.001), *IRF1* (*p* < 0.001), and *APOL6* (*p* < 0.0001) but no difference in FC of *OAS1*.

### STAT1 GOF mutants show variability in STAT1 phosphorylation, de-phosphorylation, and nuclear accumulation

**Fig. 5:**
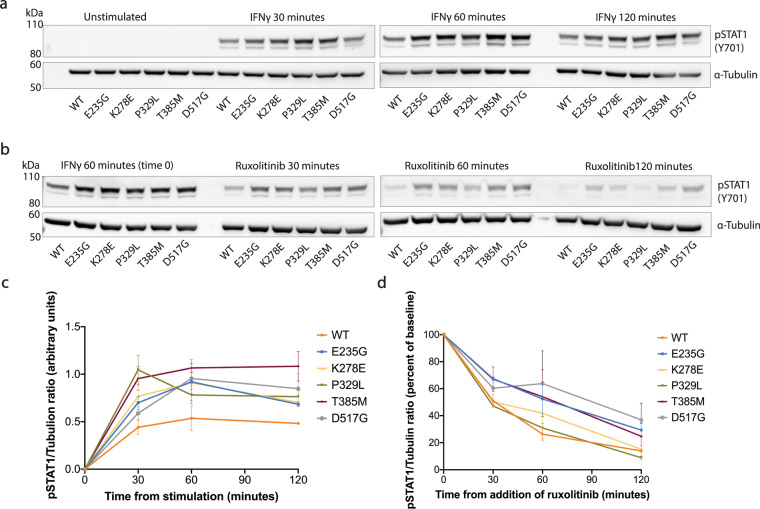
Time-course analysis of STAT1 phosphorylation and de-phosphorylation among wild type and gain-of-function mutants following IFNγ stimulation. **a** Levels of pSTAT1 were measured in whole-cell protein lysates at baseline, and following 30, 60, or 120 min of stimulation with IFNγ (10 ng/mL). Results were normalized to α-Tubulin loading control. **b** Levels of pSTAT1 were measured after 60 min of stimulation with IFNγ (10 ng/mL), and at 30, 60, or 120 min following addition of the Janus-kinase inhibitor, Ruxolitinib (10 μM). Experiments were performed in duplicates. Densitometry analysis results for the phosphorylation and de-phosphorylation assays above were graphed and are presented in panels **c** and **d**, respectively.

Given the notable transcriptional variability among GOF mutants, we evaluated whether there were differences among mutants with respect to STAT1 phosphorylation, de-phosphorylation, or nuclear accumulation. For this set of experiments, only WT and GOF mutants were evaluated. Experiments were performed in duplicates. We began by performing a time-course experiment, stimulating cells with IFNγ (10 ng/mL) for periods of 30, 60, or 120 min, following which pSTAT1 was measured by immunoblotting (Fig. 5a, Supplementary Fig. 3c and Supplementary Table 3). Overall, all cell groups reached peak pSTAT1 at 60 min after stimulation, with a slight decline noted at the 2-h time point. The exception was P329L, which peaked at 30 min and declined thereafter. At the 2-h mark, all mutants still displayed higher pSTAT1 compared with WT. Notably, the kinetics of STAT1 phosphorylation appeared somewhat slower in HAP1 cells compared with what had been previously described for primary immune cells.

Subsequently, a de-phosphorylation assay was performed by stimulating cells with IFNγ for 60 min, then adding the JAK-inhibitor, Ruxolitinib (10 μM) for 30, 60, or 120 min. The rate of pSTAT1 decline was then calculated as percent of baseline pSTAT1 (after IFNγ stimulation and prior to addition of Ruxolitinib) (Fig. 5b, Supplementary Fig. 3d and Supplementary Table 4). While some mutants (E235G, T385M, and D517G) appeared to show a slower rate of pSTAT1 decline, others showed a rate of de-phosphorylation which was comparable to WT. As an example, at the 2-h time point, mean pSTAT1 declined to 13.9% of baseline in the WT, compared with a mean of 29% for E235G, 24.7% for T385M, and 36.9% for D517G. However, these findings did not reach statistical significance. For both the phosphorylation and de-phosphorylation time-course experiments, densitometry analysis is presented in Fig. 5c, d.

**Fig. 6:**
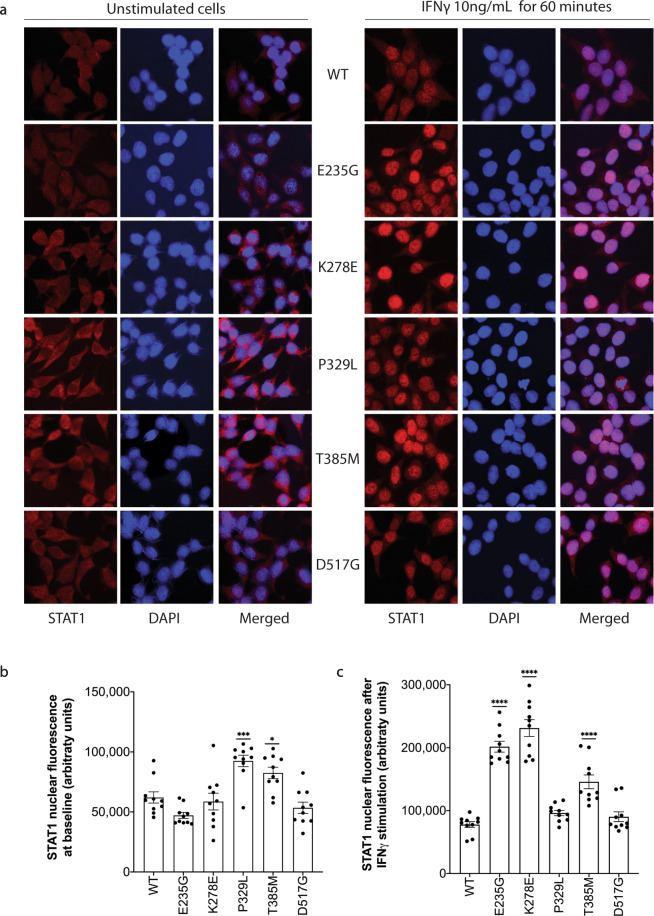
Immunofluorescence analysis of STAT1 nuclear accumulation among wild type and gain-of-function mutants. **a** Immunofluorescence assessing STAT1 nuclear localization was performed in cells at baseline (left) or following a 60-min stimulation with IFNγ (10 ng/mL). STAT1 is designated in red, while DAPI staining for nuclei is designated in blue. **b** Quantification of STAT1 from 10 nuclei per unstimulated sample. **c** Quantification of STAT1 from 10 nuclei per IFNγ-stimulated sample. Data are represented as mean ± standard error of mean. Statistical analysis: one-way ANOVA with Dunnett’s post hoc test (*p < 0.05, **p < 0.01, ***p < 0.001, ****p < 0.0001).

Next, we turned to look at nuclear accumulation of STAT1 before and after 60-min IFNγ stimulation, using immunofluorescence. Fluorescence intensity of STAT1 was quantified from 10 nuclei per group (Fig. 6 and Supplementary Tables 5 and 6). Across all genotypes, a very clear pattern of nuclear STAT1 shift was noted between the unstimulated and stimulated groups. At baseline, most groups showed comparable nuclear levels of STAT1 to WT. However, both DBD mutants, P329L and T385M, demonstrated elevated baseline STAT1 (*p* < 0.001 and *p* < 0.05, respectively). At 60 min, fluorescence intensity was significantly higher in E235G (*p* < 0.0001), K278E (*p* < 0.0001), and T385M (*p* < 0.0001), while nuclear fluorescence of STAT1 in the P329L and D517G mutants was comparable to WT.

## Discussion

The current study demonstrated for the implementation of CRISPR/Cas9 base-editing in creating heterozygous cells models of STAT1 GOF and LOF. Previously, most studies of STAT1 GOF were performed in patient samples, or in overexpression models. Work done in patient-derived samples, be they primary or immortalized cells, has provided a wealth of information regarding pathway alterations associated with STAT1 GOF. However, such samples are a limited resource necessitating access to patients, their obtaining can be invasive, and no perfectly isogenic control is available for comparison. Moreover, as sample collection is typically done after patients have already become symptomatic, it is challenging to exclude variability relating to factors such as concurrent systemic inflammation, infection, or immunosuppressive/modulatory treatments. In regard to overexpression models, the majority of studies have employed the *STAT1*-null U3 fibrosarcoma cells (and more recently, HEK293 cells on a WT background)^14,27–31^. These models have been instrumental in studying STAT1 phosphorylation and de-phosphorylation kinetics, as well as migration of STAT1 between the cytoplasm and the nucleus. However, such models are characterized by expression of *STAT1* under an exogenous promoter, and an inaccurate gene dosage. These factors considerably limit the application of overexpression models to the study of precise gene expression and signaling pathway alterations. This limitation is particularly substantial in the case of STAT1, a transcription factor which is under delicate transcriptional control, impacts the expression of other transcription factors, and in itself regulates its own expression.

Given the above limitations of overexpression models, there has been a growing interest in using genome-editing techniques to model immune disease. In regard to STAT1 GOF, Vargas-Hernandez et al.^40^ reportedly modeled two DBD mutations in natural killer cells using CRISPR/Cas9 genome-editing. However, no data regarding the specific CRISPR/Cas9 modality employed were provided. Additionally, no sequencing data were reported for the generated mutants, and it is therefore unclear whether the mutations were modeled in a homozygous, heterozygous, or compound heterozygous state (the latter often occurs when only one allele is successfully edited, while the other is cleaved without integration of a donor template resulting in LOF). Our current approach of mutant generation via base-editing offers an opportunity to model *STAT1* mutations in a heterozygous manner, and under control of the endogenous gene promoter, resulting in highly relevant cell models for dissecting the molecular pathogenesis of the disease from a transcriptional standpoint. Base-editing is efficient, quick, and enables modeling of a rapidly-expanding repertoire of point mutations^55–57^. As with most CRISPR/Cas9-based applications, the use of base-editing may be limited by the need for a protospacer adjacent motif (PAM) in close proximity to the area of interest. However, with the advent of newly engineered base editors with extended sequence recognition (such as SpG and SpRY editors used in this work), a wider array of PAMs may now be used in targeting sites for base-editing^52^. Furthermore, while base-editing was previously limited in its ability to create transversion mutations, recent works have expanded the arsenal of base editors, now allowing the generation of certain transversions in addition to transitions^58^.

The molecular hallmark of STAT1 GOF has been designated as elevated pSTAT1 (Tyr701) following type I or II IFN stimulation^14^. However, the uniformity of this finding across all patients is perplexing in the context of high clinical variability. This suggests that elevated pSTAT1 does not fully account for STAT1 GOF disease pathogenesis. The notion that pSTAT1 may be a secondary feature of STAT1 GOF has received support in recent years. For instance, some studies in patient samples found STAT1 itself to be elevated, suggesting that total STAT1, rather than pSTAT1, is the primary disease driver of STAT1 GOF^59,60^. In our current study, pSTAT1 was elevated in all GOF mutants following stimulation (as described in patients). In addition, increased total STAT1 was seen in one GOF mutant. It is possible that elevated STAT1 levels would develop across all mutants over time following repeated stimuli.

Our analysis of gene expression at baseline and following serum starvation further supports the notion of total STAT1, rather than pSTAT1, as driving the transcriptional abnormalities seen in STAT1 GOF. Our cell models showed baseline polarization in terms of ISG expression among certain mutants, suggesting that transcriptional homeostasis for some *STAT1* GOF mutants is different than that of WT. These results are recapitulated under conditions of serum starvation, suggesting that this baseline polarization may occur in a manner which is independent of external cytokine stimuli, and possibly independent (or only partially dependent) of pSTAT1. While cytokine-dependent activation of pSTAT1 has been regarded as the canonical pathway of STAT1 signaling, a well-established transcriptional role exists for unphosphorylated STAT1 (U-STAT1)^61–64^. U-STAT1 mediates the constitutive baseline activation of many ISG, in a manner which may be both cytokine-dependent and independent. It does so by acting both as a homodimer, and in complex with other transcription factors such as U-STAT2, and IRF9 (refs. ^7,61–65^). It is therefore possible that mutated, unphosphorylated STAT1 molecules may result in differential transcriptional activity at baseline, causing an abnormal pattern of ISG expression even in “naive” cells prior to cytokine stimulation. Taken together, our current findings support the notion that STAT1 GOF pathogenesis may not be fully attributed to canonical, cytokine-related pSTAT1 activation. Further work would be required to understand the mechanisms leading to baseline gene expression polarization among STAT1 GOF mutants, and what, if any, is the role of U-STAT1.

Treatment of STAT1 GOF mutants in our study with either IFNγ or IFNα has shown differential stimulation responses with a mixed pattern of increased, decreased, or similar fold change in ISG expression compared to WT. Differences were noted among WT and mutants, between IFNγ or IFNα stimulation, and also within the same genotype and stimulation group across different genes. A case in point would be T385M, which showed no baseline differences in ISG expression compared to WT, a gain of transcriptional function with respect to all genes following IFNa stimulation, and no difference (and even reduced fold change for one gene) after IFNγ treatment. P329L, which was marked by increased baseline ISG expression, demonstrated reduced responsiveness to both IFNγ and IFNα stimuli. Comparatively, E235G, which was also characterized by enhanced baseline ISG expression, showed reduced transcriptional responses to IFNγ but increased fold change following stimulation with IFNα. The notion of differential transcriptional response to stimuli in STAT1 GOF is supported by previous evidence from in vitro work done in patient cells. Kobbe et al.^66^ showed that in T cells from patients with the F172L mutation, fold change in *GBP1* expression was elevated compared to WT following IFNα, but not IFNγ stimulation, while *MIG1* fold change was elevated after treatment with either IFNα or IFNγ, but not combined treatment with both^66^. Meesilpavikkai et al.^67^ demonstrated that in T cells harboring the V653I mutation, *CXCL10* and *CD274* fold change was increased compared to WT after stimulation with IL-27, but not with IFNγ. Similar findings were reported by groups studying patient peripheral blood mononuclear cells (PBMC) assessing the expression of various other genes^68,69^.

Our study has interrogated the various patterns of STAT1 phosphorylation, de-phosphorylation, and nuclear accumulation across WT and GOF cells. As anticipated, and as previously shown by others^31^, pSTAT1 kinetics and nuclear accumulation was not uniform among STAT1 GOF mutants. Indeed, mutants varied with respect to pSTAT1 peaking, amenability to de-phosphorylation, and nuclear accumulation of STAT1. However, establishing a direct correlation between these patterns and the transcriptional variability among GOF mutants is challenging and likely multifactorial. Let us dissect, for instance, the differences between P329L and T385M, two mutations in the DBD with different transcriptional patterns. Looking at our time-course work, we note that pSTAT1 in P329L peaks early and declines relatively fast thereafter. T385M, on the other hand, has more sustained pSTAT1 elevation with possibly slower de-phosphorylation. This is further supported by the finding of elevated nuclear STAT1 in T385M but not in P329L at 60 min after stimulation. Interestingly, when assessing STAT1 nuclear accumulation at baseline, we discover that both mutants show enhanced nuclear STAT1 compared with WT (although P329L shows more prominent baseline accumulation than T385M).

Given both the overlapping and unique features of the two mutations described above, it is not surprising that their respective clinical phenotypes show both common and unique features. While both mutations are characterized by predisposition to fungal and viral infections and autoimmunity, aneurysms have only been described in T385M patients. This can perhaps be related to our finding in the T385M mutant of enhanced transcriptional responses to IFNα across all genes measured, which is not observed in P329L. Indeed, increased type I IFN activity has been implicated in the pathogenesis of human aortic aneurysms^70,71^. However, the connection between specific phenotypic features and transcriptional patters emerging from this work remains early and speculative, and requires further work. Other factors to consider and explore may include differential activation of STAT1-dependent and -independent transcription factor complexes among the various mutants, leading to differential ISG expression both at baseline and following stimulation, as well as specific gene promoter occupancy.

One important finding of our study relates to the designation of mutations as “GOF” or “LOF”. All in all, each STAT1 GOF mutant in our study showed evidence for transcriptional GOF with respect to at least one of the five genes measured compared to WT, either by means of increased baseline expression or by increased fold change following stimulation. However, some mutants, such as K278E and D517G showed more evidence for transcriptional LOF rather than GOF. The LOF mutant, Y701C, predictably showed reduced transcriptional responses to both stimuli across four of the five genes measured, with the exception of *OAS1* which is known to be co-regulated by non-STAT1-dependent transcriptional complexes^6^. Our findings in STAT1 GOF mutants suggest that the molecular designation of GOF (as it relates to tyrosine phosphorylation) may not always indicate heightened gene expression. The notion of diminished ISG transcriptional responses in some mutants is supported by recent studies reporting transcriptional LOF in STAT1 GOF. Ovadia et al.^29^ reported that compared with WT, the H629Y mutation showed either diminished or unchanged fold increase in gene expression in response to IFNγ. More recently, work done in a mouse model of the R274Q mutation demonstrated a reduction in the expression of the ISG *Cxcl10* and *Irf1* following viral infection in vivo^72^.

The current study is constrained by a few limitations. These include a relatively small number of genes tested, and the measurement of fold change following single stimulation of naïve cells. Future work will involve larger-scale gene studies, including pathways that extend beyond the immediate group of IFN-response genes. In regard to stimulation of naive cells, previous work showed that STAT1 GOF cells had an impaired transcriptional response not only upon initial stimulation, but also to re-stimulation^45^. It would therefore be important in the future to study how the transcriptional responses change over time and following repeated or different stimuli. Such work may further help understand the evolution of transcriptional responses as they occur in vivo. Further work may also be merited with regard to mechanisms resulting in differential gene expression across mutants and stimuli, and in particular elucidating the possible contribution of total or U-STAT1 to the abnormal gene expression patterns in STAT1 GOF. Finally, as in most research involving cell lines, the current work does not fully reflect that different roles and full breadth of activity that STAT1 may play in either immune or non-immune cells in vivo. Indeed, gene expression patterns in vivo may vary among different cell types. While the strength of this work is in uniformly comparing the impact of different mutations on the same cell type, complementation of this work by future in vivo studies in a variety of tissues is warranted.

In conclusion, we present a series of heterozygous *STAT1* cell models generated using CRISPR/Cas9 base-editing, showing the utility of this technique in modeling heterozygous immune-mediated disease. Our cell models demonstrate intricate patterns of ISG expression, involving transcriptional abnormalities at baseline, following serum starvation, and after stimulation with type I or II IFN. Taken together, our findings are in line with a growing body of literature suggestive of complex and multifactorial transcriptional responses in STAT1 GOF, which cannot be simply and generally classified as either gain or loss of function. Moreover, our findings may indicate an important role for total and U-STAT1 in disease pathogenesis, in addition to the role of elevated pSTAT1. Continued investigation of gene expression patterns associated with *STAT1* mutations may enhance our understanding of both disease pathophysiology and genotype–phenotype correlation in STAT1 GOF. This, in turn, has the potential to improve our prognostic capacity of patients affected by this disorder, and may ultimately open up new avenues for disease interrogation and targeting.

## Methods

### Mutation selection

Previously published mutations were selected for modeling according to the following criteria: (1) transition mutations (A•G or C•T) for generation by CRISPR/Cas9 base-editing; (2) patients carrying the mutations met clinical criteria for STAT1 GOF diagnosis; and (3) previously published in vitro analysis confirmed the presence of elevated Tyr701 phosphorylated STAT1 (pSTAT1) following IFN stimulation. An additional heterozygous LOF transition mutation, Y701C, was chosen for modeling as well.

### Cell culture

HAP1 cells (kind gift of Dr. Aleixo Muise, Toronto) were cultured in IMDM medium (Wisent Bioproducts 319-105-CL) supplemented with 10% heat-inactivated fetal bovine serum (FBS) (Wisent Bioproducts 080-150) and 1% penicillin–streptomycin (Wisent Bioproducts 450-201-EL). For serum starvation experiments, HAP1 cells were placed in IMDM containing 0.25% FBS and 1% penicillin–streptomycin. Fibroblasts (ATCC PCS-201–012) for DNA-content analysis were cultured in DMEM medium (Wisent Bioproducts 319-005-CL) supplemented with 10% heat-inactivated FBS and 1% penicillin–streptomycin. Cell line authentication was done by means of short tandem repeat (STR), performed by The Hospital for Sick Children, The Centre for Applied Genomics (TCAG). Cells were confirmed to be mycoplasma-free using a PCR Mycoplasma Detection Kit (Applied Biological Materials Inc. G238). Cells were cultured in a 37 °C humidified incubator containing 5% CO_2._

### DNA-content analysis

DNA-content analysis was performed as previously described^73^, comparing HAP1 cells and diploid fibroblasts. Briefly, cells were washed with 1× phosphate-buffered saline (PBS) (Wisent Bioproducts 311-010-CL), trypsinized, and resuspended in complete media (1 × 10^6^ cells/µl), to which a low-toxicity, cell-permeable DNA dye (Vybrant DyeCycle Violet^TM^; 1:1000, Thermo Fisher Scientific V35003) was added. Cells were incubated at 37 °C for 30 min. Live/dead cell stain was concurrently performed using propidium iodide (1 µg/µL; Thermo Fisher Scientific P1304MP), added according to the manufacturer’s recommendation. Samples were run on BD LSR-II^TM^ with the BD FACSDiva^TM^ software v9.0, using the services of the Hospital for Sick Children Flow Cytometry Facility. Data were analyzed using FlowJo version 10.7.1. MFI peaks were compared for HAP1 and fibroblasts at G_0_/G_1_ and G_2/_M.

### Oligonucleotides and primers

Information regarding oligonucleotides and primers used in this work is provided in Table 4.

**Table 4:** Oligonucleotides and primers used in this work.

Name	Sequence
E235G_cloning_top	5′-CACCGATGAACTAGTGGAGTGGAAG-3′
E235G_cloning_bottom	5′-AAACCTTCCACTCCACTAGTTCATC-3′
K278E_cloning_top	5′-CACCGCTTAAAAAGTTGGAGGAAT-3′
K278E_cloning_bottom	5′-AAACATTCCTCCAACTTTTTAAGC-3′
P329L_cloning_top	5′-CACCGCACCCTCAGAGGCCGCTGGT-3′
P329L_cloning_bottom	5′-AAACACCAGCGGCCTCTGAGGGTGC-3′
T385M_cloning_top	5′-CACCGGGCACGCACACAAAAGTGA-3′
T385M_cloning_bottom	5′-AAACTCACTTTTGTGTGCGTGCCC-3′
D517G_cloning_top	5′-CACCGTGTGGACCAGCTGAACATGT-3′
D517G_cloning_bottom	5′-AAACACATGTTCAGCTGGTCCACAC-3′
Y701C_cloning_top	5′-CACCGTGGATATATCAAGACTGAGT-3′
Y701C_cloning_bottom	5′-AAACACTCAGTCTTGATATATCCAC-3′
E235G_Amp_F	5′-CATGGGTCACTGAAAACAAGT-3′
E235G_Amp_R	5′-GCCAGTTTTCTGCTTTGGAG-3′
K278E_Amp_F	5′-TTGTTGGTTTCCATGCCATA-3′
K278E_Amp_R	5′-GGAGGATTGCTTGAACTTGG-3′
P329L_ Amp_F	5′-CCCACTTCAACCCTCCAGTA-3′
P329L_ Amp_R	5′-GGGGTTCATAAGGCTCAGGT-3′
T385M_ Amp_F	5′-CAATGTAAGGCCCAGACCAT-3′
T385M_ Amp_R	5′-ACCCTGGTGTACAGGACCAC-3′
D517G_ Amp_F	5′-TCGAATTCTTTGCTGCTGTG-3′
D517G_ Amp_R	5′-GCAAGCCCCAGGACTTTATT-3′
Y701C_ Amp_F	5′-GCCAGGCTAATGCCAATAAA-3′
Y701C_ Amp_R	5′-TGCAGGCCAAATAACTGACA-3′
E235G_Seq	5′-TGACCTGTCACTAGGCAGCA-3′
K278E_Seq	5′-TGTGACTTTGCTCCTCATTTG-3′
P329L_Seq	5′-TCCCTATTAGGTTTTGGGATTTC-3′
T385M_Seq	5′-TGCAGAGATGTGAATGAGAGAAA-3′
D517G_Seq	5′-AGTGCCACACTTGTGACTGG-3′
Y701C_Seq	5′-TCTCGTTGTTTCTGCATTCC-3′
GBP1_qPCR_F	5′-AGGAGTTAGCGGCCCAGCTAGAAA-3′
GBP1_qPCR_R	5′-AAAATGACCTGAAGTAAAGCTGAGC-3′
IFIT2_qPCR_F	5′-GCACTGCAACCATGAGTGAGA-3′
IFIT2_qPCR_R	5′-CAAGTTCCAGGTGAAATGGCA-3′
IRF1_qPCR _F	5′-TCCTGCAGCAGAGCCAACATGCCCA-3′
IRF1_qPCR _R	5′-CCGGGATTTGGTTGGAATTAATCTG-3′
APOL6_qPCR_F	5′-TTGGTTTGCAAAGGGATGAGGATGA-3′
APOL6_qPCR _R	5′-TCTTTCAATCTGGGAAATTCTCTCA-3′
OAS1_qPCR_F	5′-CAAGGTGGTAAAGGGTGGCTCCTCA-3′
OAS1_qPCR _R	5′-TAACTGATCCTGAAAAGTGGTGAGA-3′
GAPDH_qPCR_F	5′- CAATGACCCCTTCATTGACCTC-3′
GAPDH_qPCR R	5′- GATCTCGCTCCTGGAAGATG -3′

### Cloning

The sgRNA vector, BPK1520_puroR, a plasmid containing a cloning site for sgRNA under control of a U6 promoter, as well as a puromycin resistance cassette, was generated as previously described^35^. Cloning oligonucleotides were annealed using an annealing buffer (10 mM Tris, pH 7.5–8.0, 50 mM NaCl, 1 mM EDTA) and phosphorylated using T4 Polynucleotide Kinase (New England BioLabs M0201L) according to the manufacturer’s recommendations. BPK1520_puroR was linearized with BsmBI (New England BioLabs R0580S) and dephosphorylated using recombinant shrimp alkaline phosphatase (New England BioLabs M0371L). Annealed and phosphorylated oligonucleotides were cloned into linearized and dephosphorylated BPK1520_puroR using T4 DNA Ligase (New England BioLabs M0202L) according to the manufacturer’s recommendations. Subsequently, One Shot™ TOP10 Chemically Competent *E. coli* (Thermo Fisher Scientific C404003) were transformed with the ligation products and plated on LB-Ampicillin agar plates (50 µg/mL ampicillin). Resultant colonies were inoculated overnight in LB-ampicillin, and plasmids purified using the QIAprep Spin Miniprep Kit (Qiagen 27106) according to the manufacturer recommendations. The following plasmids were used for the purpose base-editing: pCMV_ABEmax_P2A_GFP (gift from Dr. David Liu, Addgene plasmid 112101)^51^ was used to generate E235G, K278E, and D517G. Plasmids pCAG-CBE4max-SpRY-P2A-EGFP, pCAG-CBE4max-SpG-P2A-EGFP and pCMV-T7-ABEmax(7.10)-SpRY-P2A-EGFP (gifts from Dr. Benjamin Kleinstiver, Addgene plasmids 13999, 139998 and 140003)^52^ were used to generate P329L, T385M and Y701C, respectively.

### Transfection and selection

Twenty-four hours prior to transfection, 4 × 10^5^ cells were seeded in a 12-well plate. The following day, cells were transfected with 1250 ng of total DNA, containing the Cas9 base-editor expression vector and the sgRNA expression vector containing the PuroR gene, at a 1:1 ratio (w/w). Transfection was performed using Lipofecatmine^TM^ 3000 Transfection Reagent (Thermo Fisher Scientific L3000001) according to the manufacturer’s recommendations. To enrich for transfected cells, 24 h post-transfection cells were subjected to puromycin selection (0.7 µg/mL; Thermo Fisher Scientific A1113803) for 72 h. Following puromycin selection, estimation of base-editing efficiency in the bulk population was performed as follows: 1 × 10^6^ cells were collected from which genomic DNA was isolated using the DNeasy Blood & Tissue Kit (Qiagen 69506). This was followed by PCR amplification of the desired region using DreamTaq Polymerase (Thermo Fisher Scientific EP0705). Amplified DNA was PCR-purified using QIAquick PCR Purification Kit (Qiagen 28106) following the manufacturer’s protocol and prepared for Sanger sequencing using the BigDye™ Terminator v3.1 Cycle Sequencing Kit (Thermo Fisher Scientific 4337457). Samples were sequenced on an Applied Biosystems SeqStudio Genetic Analyzer (Thermo Fisher Scientific), and sequencing AB1 files were input into the online base-editing analysis tool, editR^74^. This provided an estimated percentage editing of the bulk population, as well as percentage editing (if any) of any adjacent bases.

### Single-cell sorting and clone screening

Following estimation of editing efficiency, cells were trypsinized and resuspended in FACS buffer (1× PBS without calcium and magnesium pH 7.4, supplemented 2% FBS, and 2.5 mM EDTA) at a concentration of 1 × 10^6^ cells/mL. Live/dead cell stain was performed using propidium iodide. Live single cells were sorted on a MoFloXDP Cell Sorter (Beckman Coulter), using the services of the Hospital for Sick Children Flow Cytometry Facility. Cells were sorted into a 96-well plate containing full media and allowed to clonally expand for a period of 14 days. Following a 2-week recovery period, single-cell clones underwent genomic DNA isolation, followed by PCR amplification, purification, and Sanger sequencing as described above.

### IFNγ stimulation and determination of STAT1 phosphorylation and de-phosphorylation

Determination of total STAT1 and pSTAT1 protein levels in IFNγ-stimulated or unstimulated cells was done over five independent experiments. For each experiment, 1 × 10^6^ cells were seeded in a six-well plate. Twenty-four hours later, Human Recombinant IFNγ (10 ng/mL, StemCell Technologies 78020) was added to the media for a period of 60 min. Following stimulation, cells were washed with cold PBS and immediately harvested. Subsequently, we investigated the phosphorylation and de-phosphorylation kinetics of pSTAT1 following IFNγ stimulation, with each experiment performed in duplicates. Cells were left unstimulated, or stimulated with IFNγ for 30, 60, or 120 min before being harvested. Finally, we evaluated the de-phosphorylation of pSTAT1 across cell groups following the addition of the JAK-inhibitor, Ruxolitinib (Selleckchem, S1378). To this end, cells were stimulated with IFNγ for 60 min, following which Ruxolitinib (10 μM final concentration) was added for 30, 60, and 120 min prior to cell harvesting.

### Immunoblotting

Immunoblotting was used to determine STAT1 and/or pSTAT1 protein levels. Following stimulation experiments as above, whole-cell lysates were obtained by lysing cells in RIPA Lysis and Extraction Buffer (Thermo Fisher Scientific 89900), supplemented with Halt™ Protease and Phosphatase Inhibitor Cocktail (Thermo Fisher Scientific 78440), on ice for 30 min. Lysates were sonicated and then centrifuged at 12,000 × *g* for a period of 15 min at 4 °C. Protein concentrations were determined using the Pierce™ BCA Protein Assay Kit (Thermo Fisher Scientific 23552). Samples were then prepared by addition of NuPAGE™ LDS Sample Buffer (4×) (Thermo Fisher Scientific NP0007) followed by boiling at 100 °C for 5 min. Samples were subjected to SDS-Page separation by running 20 µg of total protein on a NuPage 4–12% Bis–Tris gel (Thermo Fisher Scientific NP0336BOX) using NuPAGE™ MOPS SDS Running Buffer (Thermo Fisher Scientific NP000102). For each experiment, samples for pSTAT1 and STAT1 were run in parallel. Protein was subsequently transferred to a nitrocellulose membrane using the iBlot 2 Dry Blotting System (Thermo Fisher Scientific). Following transfer, membranes were blocked in 1× Tris-buffered saline (50 mM Tris-Cl, pH 7.5. 150 mM NaCl) containing 5% bovine serum albumin (Sigma Aldrich A7906-50G) for 1 h at room temperature. Membranes were then incubated at 4 °C overnight with primary antibodies against pSTAT1 [(pY701), 1:500; clone D4A7; Cell Signaling 7649S)], total STAT1 (1:500, Clone D1K9Y; Cell Signaling 14994) or alpha-tubulin (1:1000, Clone DM1A; Sigma Aldrich T6199–100UL). The following day, membranes were washed in tris-buffered saline and incubated for 1 h at room temperature with one of the following secondary antibodies: Donkey anti-Rabbit IgG (H + L) Highly Cross-Adsorbed Secondary Antibody, Alexa Fluor 647 (1:1000; Thermo Fisher Scientific A-31573) or Donkey anti-Mouse IgG (H + L) Highly Cross-Adsorbed Secondary Antibody, Alexa Fluor 647 (1:2500; Thermo Fisher Scientific A-31571). Membranes were imaged using ChemiDoc MP imaging system (Bio-Rad) and analyzed with Image Lab software (^©^2017 Bio-Rad Laboratories; version 6.0.1). All blots presented together in this work were derived from the same experiments and were processed in parallel.

### Immunofluorescence

Nuclear accumulation of STAT1 following 60 min of IFNγ stimulation was determined by immunofluorescence. Cells were seeded in a 24-well plate containing coverslips at 2 × 10^5^ cells/well. The next day, following stimulation with IFNγ as noted above, cells were fixed and permeabilized using ice-cold methanol and kept at −20 °C for 20 min. Cells were subsequently thoroughly washed with PBS, and kept in blocking solution (5% FBS in PBST) for an hour. Cells were then incubated overnight at 4 °C in primary anti-STAT1 antibody (1:200; Clone D1K9Y; Cell Signaling 14994). The following day, cells were thoroughly washed in PBST and incubated in secondary antibody (1:1000, Goat anti-Rabbit IgG (H+L) Cross-Adsorbed Secondary Antibody, Alexa Fluor 555; Thermo Fisher Scientific A-21428) for an hour at room temperature. Nuclear staining was done using DAPI (1:1000; Thermo Fisher Scientific 62248). Cells were finally thoroughly washed, dried, mounted on slides, and imaged using Leica SP8 Lightning Confocal Microscopy. Acquired images were analyzed using ImageJ version 2.1.0/1.53c. For each group, fluorescence from 10 nuclei was quantified.

### RNA isolation and quantitative real-time PCR

RNA analysis was performed to determine gene expression levels across the different genotypes under various conditions. Experiments were repeated a minimum of five times for each gene, in at least technical duplicates, and pooled data for each gene was collected. To determine baseline gene expression levels, cells were grown in full media and harvested without stimulation once reaching 70–80% confluence. For determination of baseline gene expression under low-serum conditions, cells were seeded as described above and allowed to adhere in full media for a period of 24 h. Cells were subsequently thoroughly washed with PBS and placed in low-serum media (IMDM + 0.25% FBS) for an additional 24 h. For stimulation experiments, cells in full media were incubated with Human Recombinant IFNα-2A (10 ng/mL; StemCell Technologies 78076.1) or IFNγ (10 ng/mL) for 6 h prior to harvesting and RNA extraction using RNeasy Mini Kit (Qiagen 74106). Next, 1000 ng of RNA was reverse-transcribed using SuperScript™ III First-Strand Synthesis System (Thermo Fisher Scientific 18080051) following the manufacturer’s protocol. Quantitative real-time PCR (qRT-PCR) using PowerUp™ SYBR™ Green Master Mix (Thermo Fisher Scientific A25742) was performed on an Applied Biosystems QuantStudio 3 Real-Time PCR System (Applied Biosystems). Quantification of the following genes was done: *GBP1, IFIT2, IRF1, APOL6, OAS1*, with *GAPDH* used as housekeeping control. The relative expression levels were compared using the ΔΔCt method.

### Statistical analysis

Graphical data were represented as means ± standard error of mean. Statistical analysis was performed using GraphPad Prism 8 (version 8.4.3). One-way ANOVA with Dunnett’s post hoc test was used to determine differences among mutants and wild type. Statistical significance was represented as: *p < 0.05, **p < 0.01, ***p < 0.001, ****p < 0.0001.

### Reporting summary

Further information on research design is available in the Nature Research Reporting Summary linked to this article.

## Supplementary information

Supplementary Information

Reporting Summary

## Data Availability

The data that support the findings of this study are available from the corresponding author upon reasonable request.
