# Supplementary material for: STAT1 gain-of-function heterozygous cell models reveal diverse interferon-signature gene transcriptional responses
Source: NPJ Genom Med. 2021 May 14;6:34. doi: 10.1038/s41525-021-00196-7 (PMC8121859; doi:10.1038/s41525-021-00196-7)
Supplement: Supplementary file 1 — Supplementary Information [file 41525_2021_196_MOESM1_ESM.pdf]

Supplementary Material

Supplementary Figure Legends: pages 2-3
Supplementary Figures: pages 4-13
Supplementary Tables: pages 14-29

Supplementary Figure Legends

Supplementary Figure 1. DNA content analysis demonstrates diploidy of HAP1 cells. | (a) DNA content was measured in HAP1 (blue), wildtype human fibroblasts (known to be diploid; orange) and a 1:1 mixture of fibroblasts and HAP1 cells (red). DNA content was assessed by measuring the median fluorescence intensity (MFI) of a live cell DNA dye (Vybrant DyeCycle Violet^{TM}) across the cell cycle. MFI ratios between HAP1 and fibroblasts were calculated for the G_0/G_1 phase (left peak) and G_2/M phase (right peak). An MFI ratio of ~1 for both peaks indicate that the ploidy of HAP1 cells is identical to that of diploid fibroblasts. **(b)** Gating strategy used for DNA content analysis. Following exclusion of debris in the FSC-A/SSC-A window, doublets were excluded using FSC-A/FSC-H followed by the SSC-A/SSC-H parameters. Dead cells were excluded by plotting the G660-A (propidium iodide) fluorescence intensity vs. SSC-H. Events associated with G660-A fluorescence intensity higher than 10^3 were excluded. Finally, for all live single cells, fluorescence intensity ofV450-A (Vybrant DyeCycle Violet^{TM}) was plotted as a histogram.

Supplementary Figure 2. workflow for generating and verifying *STAT1* mutants. | HAP1 cells were seeded in a 12-well plate. Twenty-four hours later, cells were transfected by lipofection with plasmids containing the single-guide RNA (sgRNA) and the Cas9 base-editor, as well as a puromycin-resistance cassette. Following puromycin selection, cells were single-cell sorted into 96-well plates and clones. Following a 2-week expansion period, DNA was extracted from a sample of cells in each well, and genotyped by Sanger sequencing.

Supplementary Figure 3. Uncropped immunoblot membranes presented in main manuscript. | (a) Top: expression of pSTAT1 (Tyr701) compared with α-tubulin loading control, following 60 minutes of stimulation with IFNγ (10ng/mL), using long-exposure at time of imaging; Bottom: the same membrane as above, imaged using short-exposure. **(b)** Total STAT1 compared with α-tubulin loading control following 60 minutes of stimulation with IFNγ (10ng/mL). **(c)** pSTAT1 time-course at 0, 30, 60 and 120 minutes following stimulation with IFNγ (10ng/mL), compared with α-tubulin loading control. **(d)** pSTAT1 de-phosphorylation assay: pSTAT1 expression following stimulation with IFNγ (10ng/mL) at 0, 30, 60 and 120 minutes following Ruxolitinib administration. Note: as fluorescent imaging may introduce artifact to protein ladders, colorimetric images of ladders for all blots are provided as well for each blot.

Supplementary Figure 4. STAT1-associated gene transcription under various experimental conditions. | **(a)** at baseline, constitutive Interferon-Stimulated Gene (ISG) expression is maintained at a basal level in an interferon (IFN)-independent manner via two unphosphorylated transcription complexes: unphosphorylated gamma activating factor (U-GAF) made up of two STAT1 units, and unphosphorylated interferon-stimulated gene factor 3 (U-ISGF3), made up of STAT1, STAT2 and IRF9. At baseline, mammalian target of rapamycin (mTOR) serves as an inhibitor of STAT1 migration to the nucleus, preventing excessive ISG transcription; **(b)** following nutrient depletion, mTOR is inhibited, allowing for higher nuclear accumulation unphosphorylated-STAT1 containing transcriptional complexes. This results in an ISG transcriptional response which is IFN-independent; **(c)** Stimulation with IFNα begins with ligation of the type I IFN receptor, made up of two subunits: IFNAR1 and IFNAR2. Receptor

ligation leads to recruitment of the tyrosine kinases TYK2 and JAK1, which cross-phosphorylate each other as well as the intracellular receptor domains. This creates a docking site for STAT1 and STAT2 which bind, are phosphorylated, and along with IRF9 form the transcription complex ISGF3. ISGF3 migrates to the nucleus where it binds to interferon-stimulated response element (ISRE) in gene promoters, resulting in a Type I IFN response; **(d)** Stimulation with IFNγ begins with ligation of the type II IFN receptor, made up of two subunits each of IFNGR1 and IFNGR2. Receptor ligation leads to recruitment of the tyrosine kinases JAK1 and JAK2, which cross-phosphorylate each other as well as the intracellular receptor domains. This creates a docking site for STAT1 which bind, are phosphorylated, and form the homodimer GAF. GAF migrates to the nucleus where it binds to gamma-activating sequence (GAS) in gene promoters, resulting in a Type II IFN response.

Supplementary Figures
Supplementary Figure 1:

a

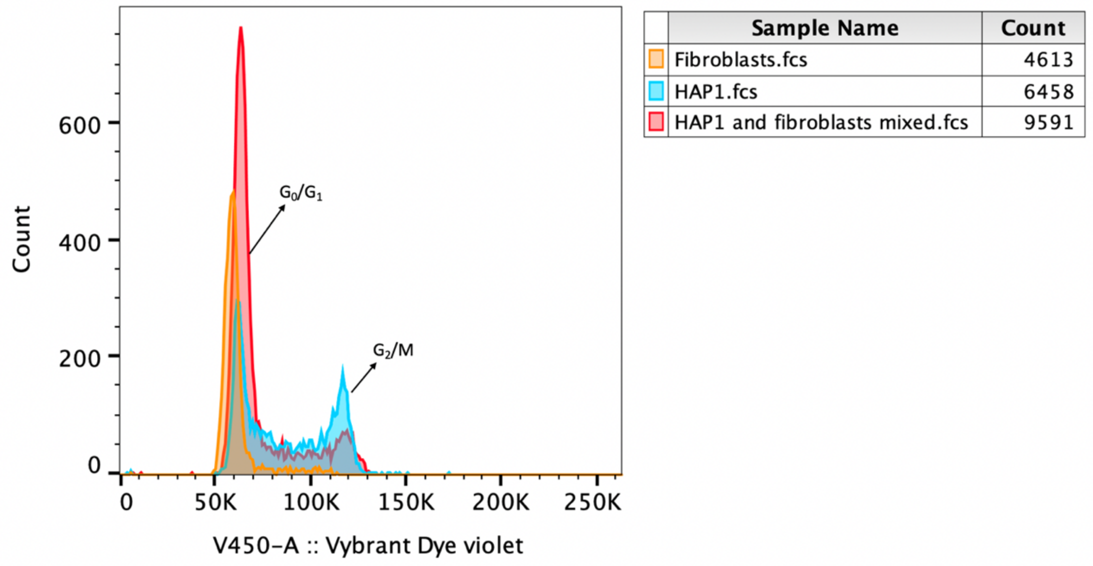

b

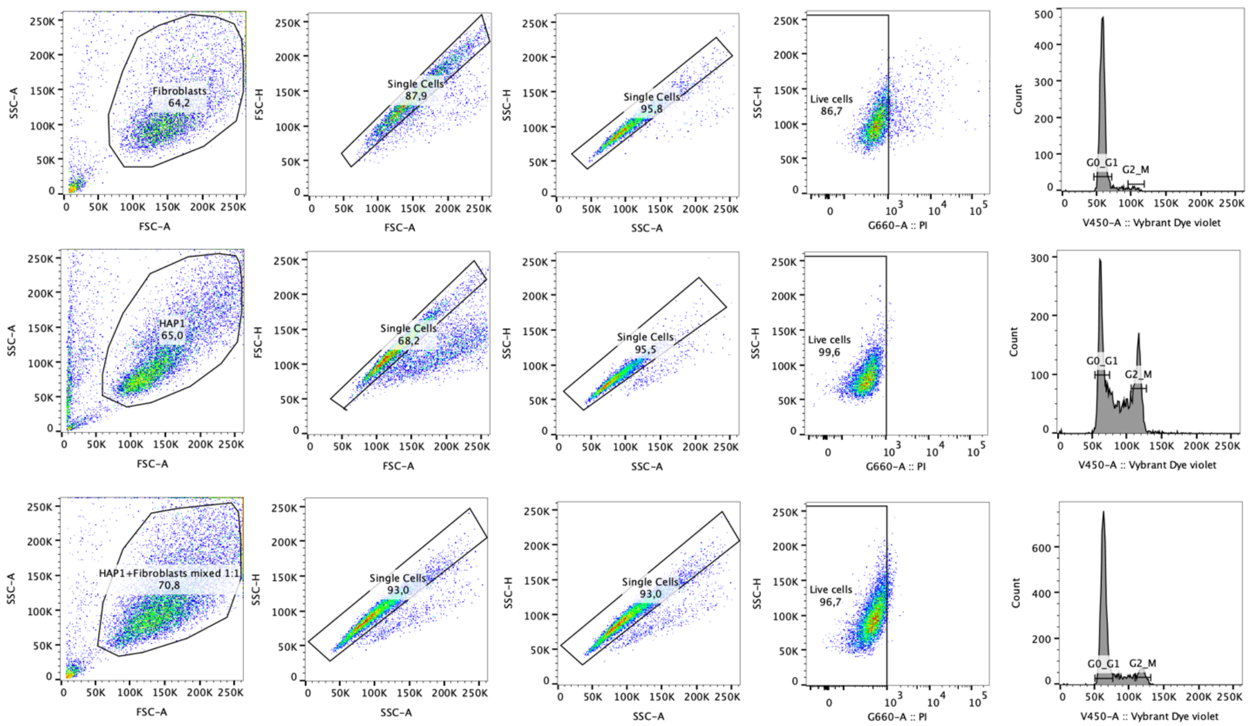

Supplementary Figure 2:

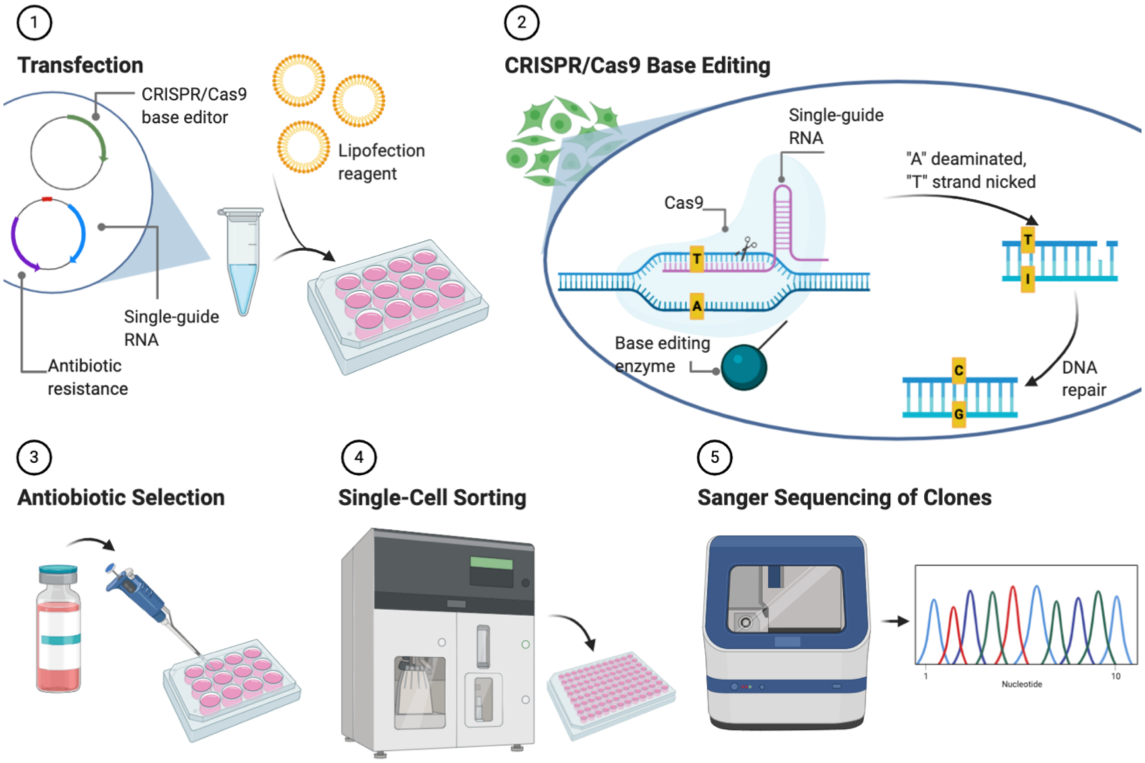

Supplementary Figure 3:
(a)
Long-exposure:

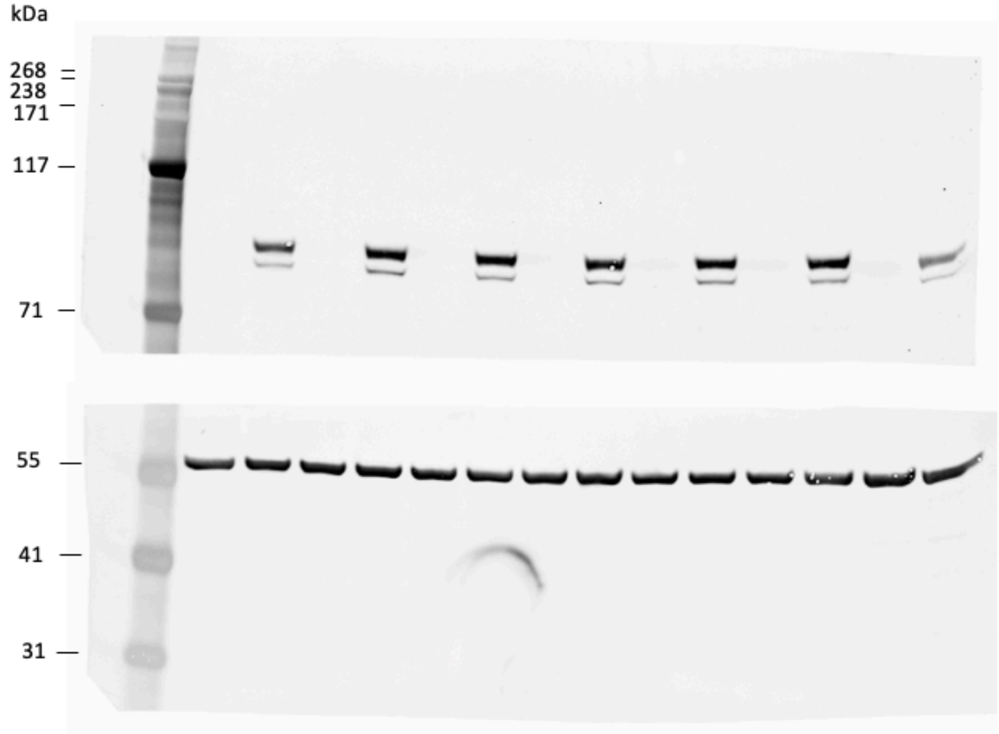

Short-exposure:

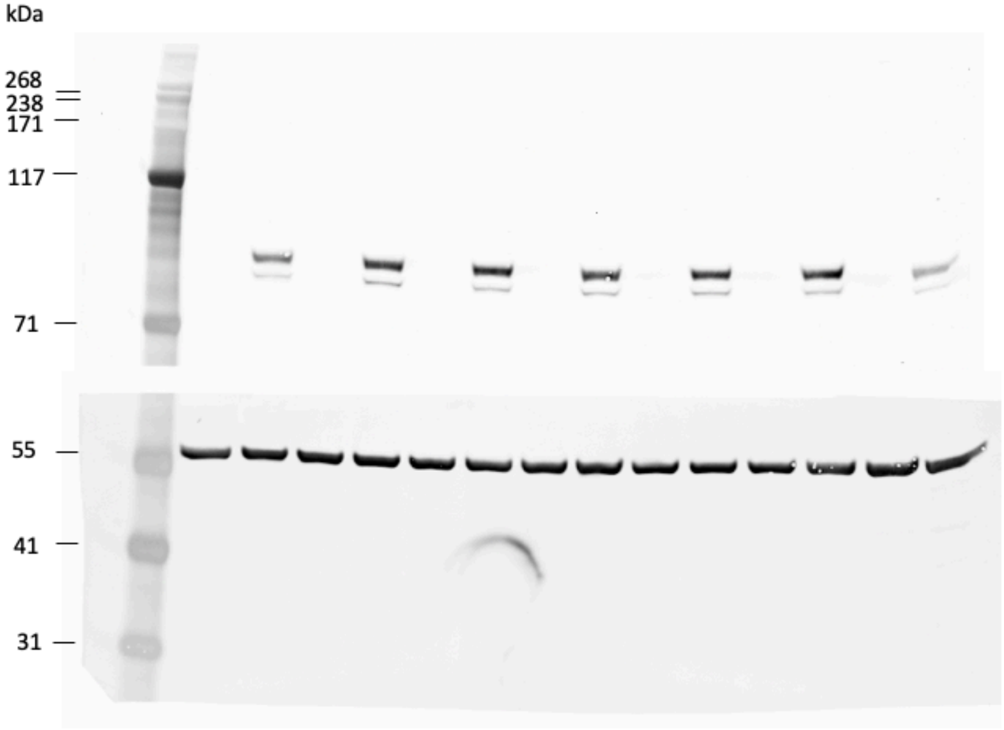

Colorimetric ladder image:

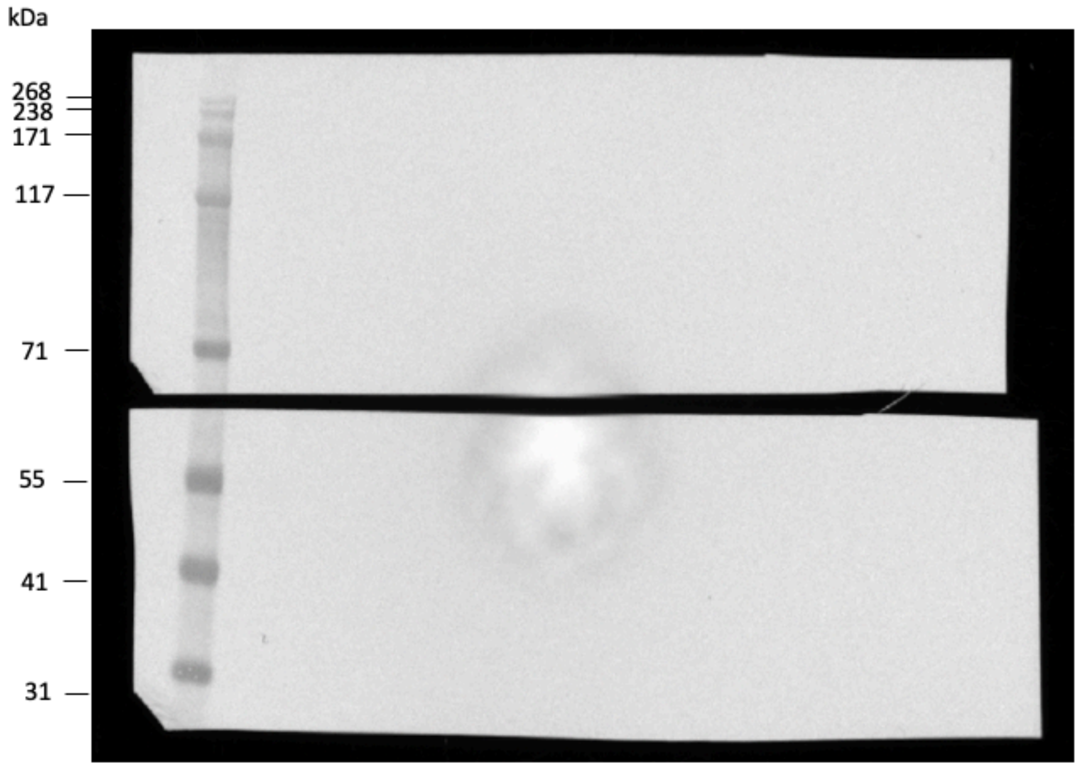

(b)

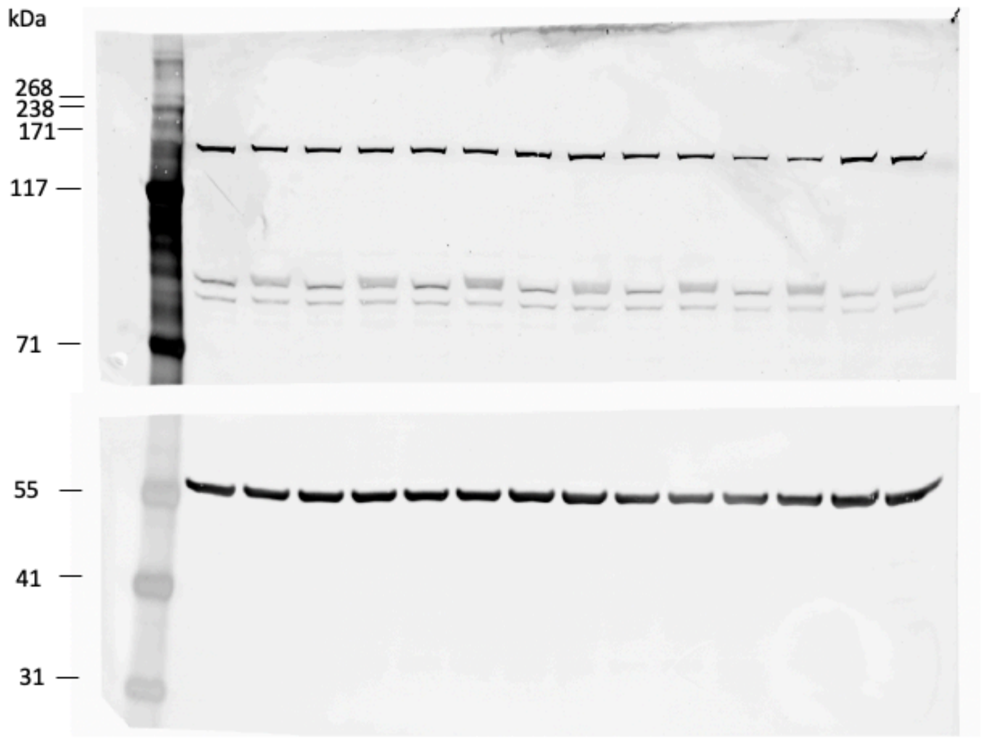

Colorimetric ladder image:

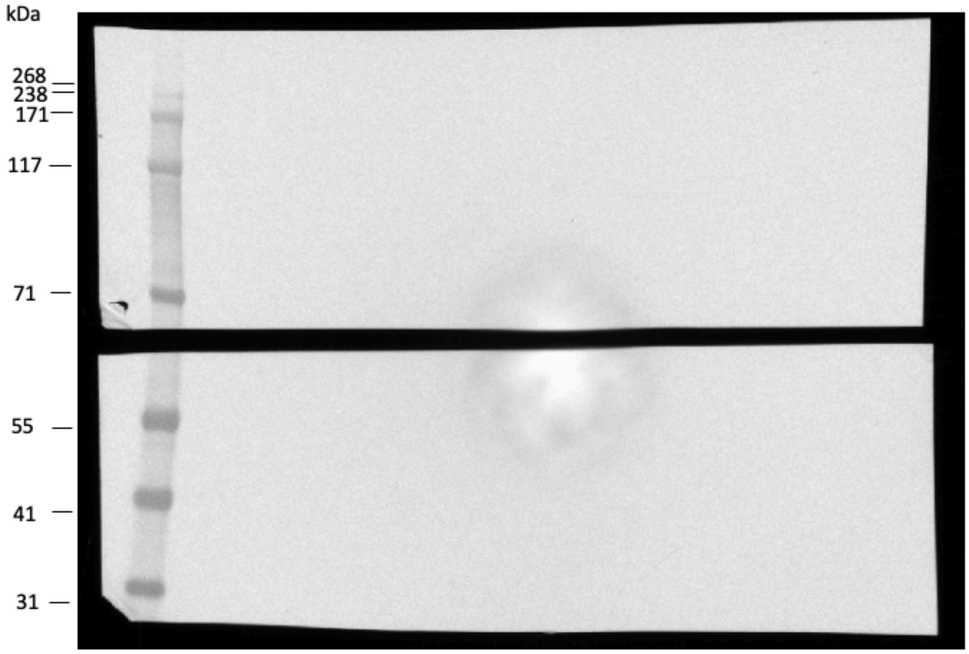

(c)
0-30 minutes:

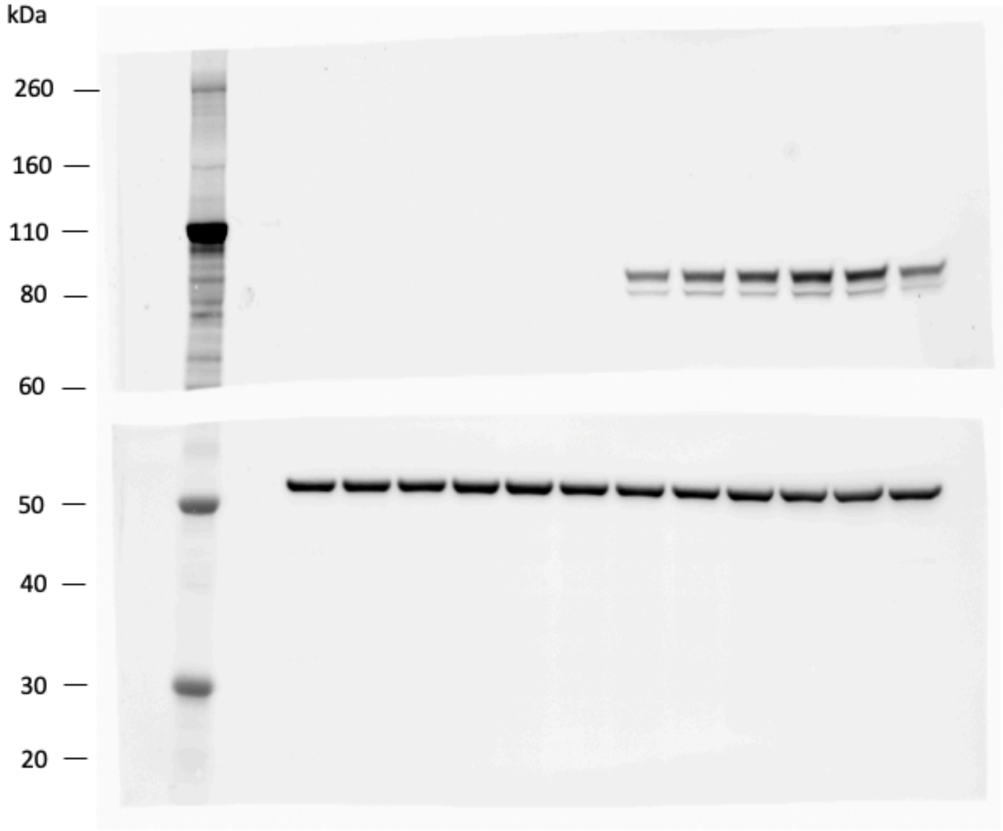

Colorimetric ladder image:

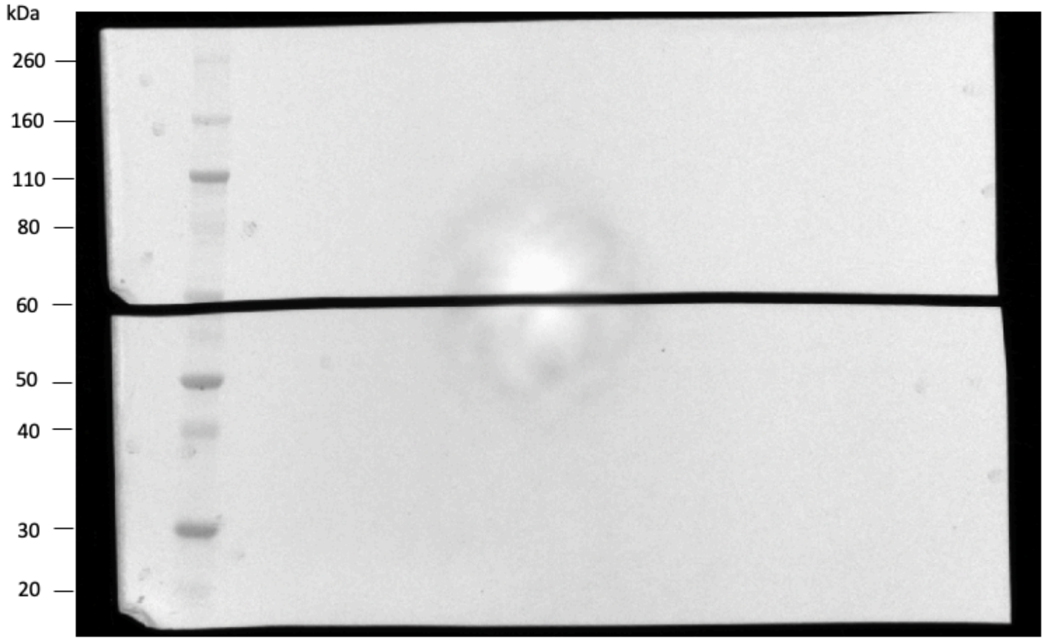

60-120 minutes:

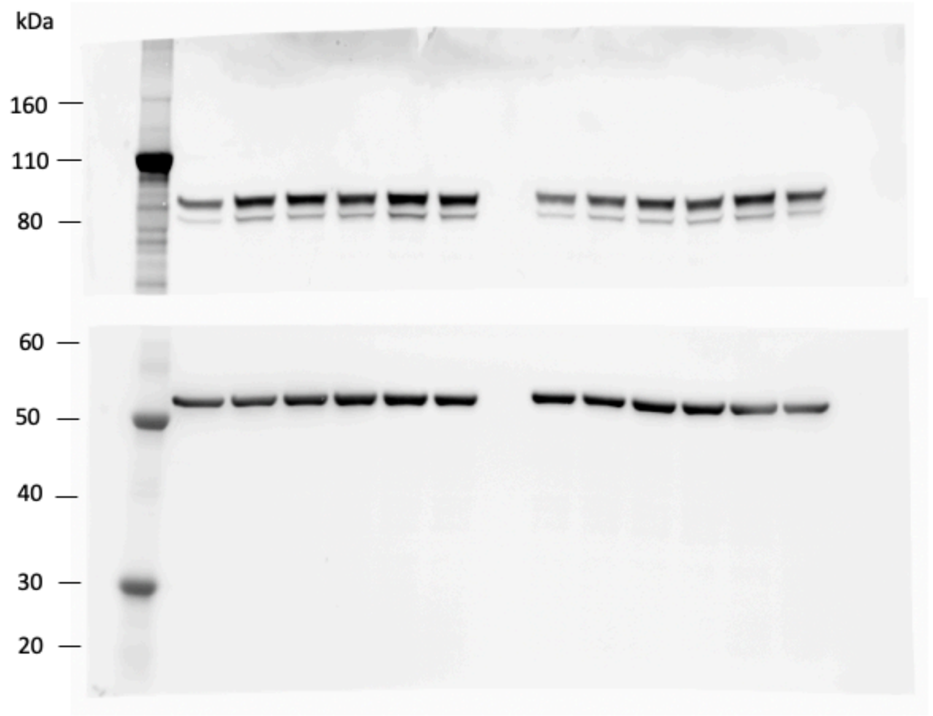

Colorimetric ladder image:

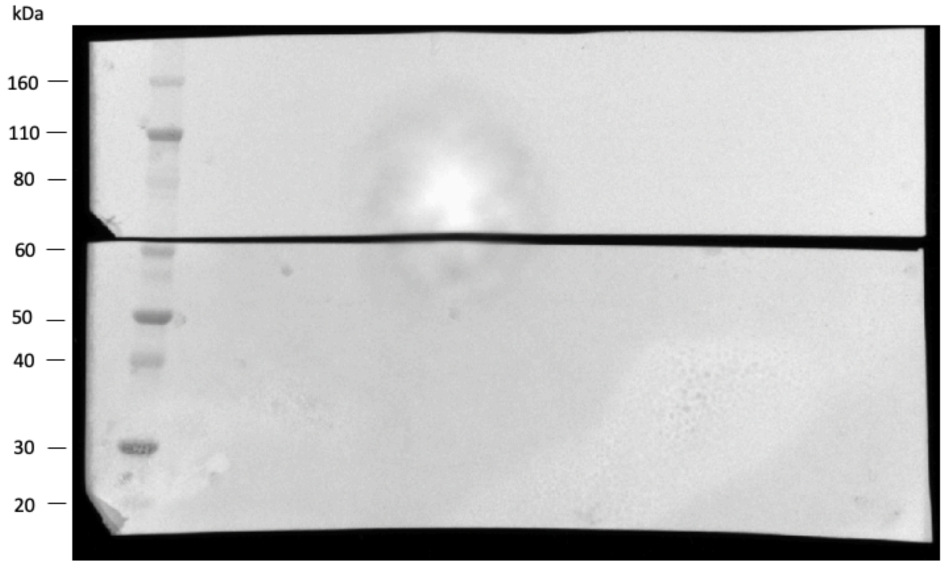

(d)

0-30 minutes:

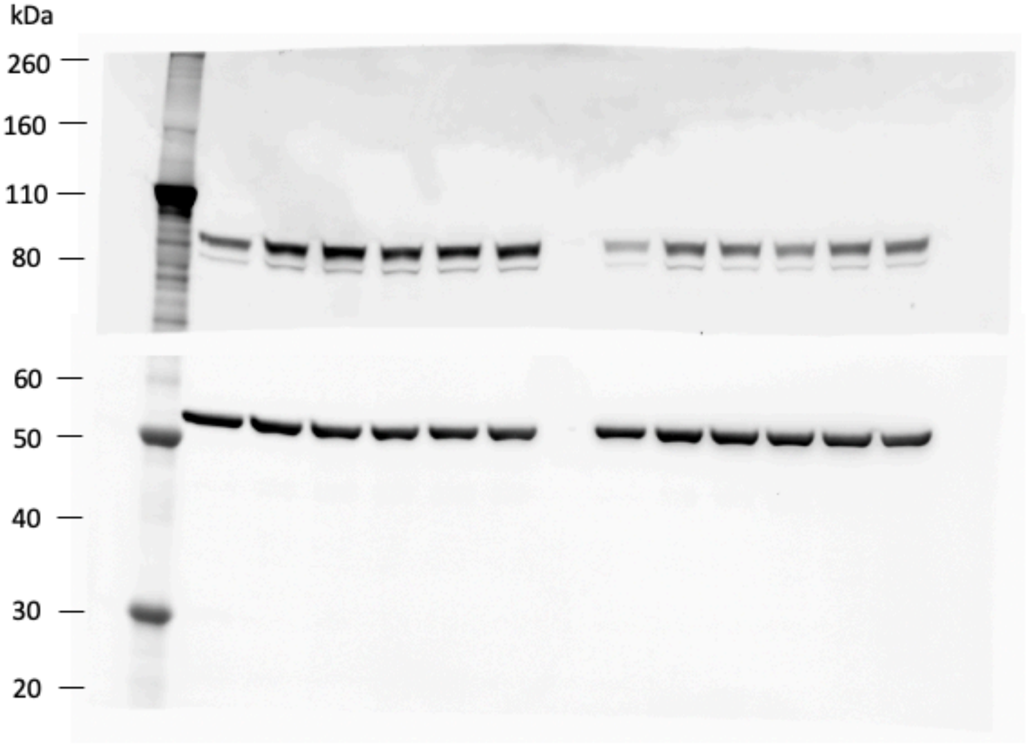

Colorimetric ladder image:

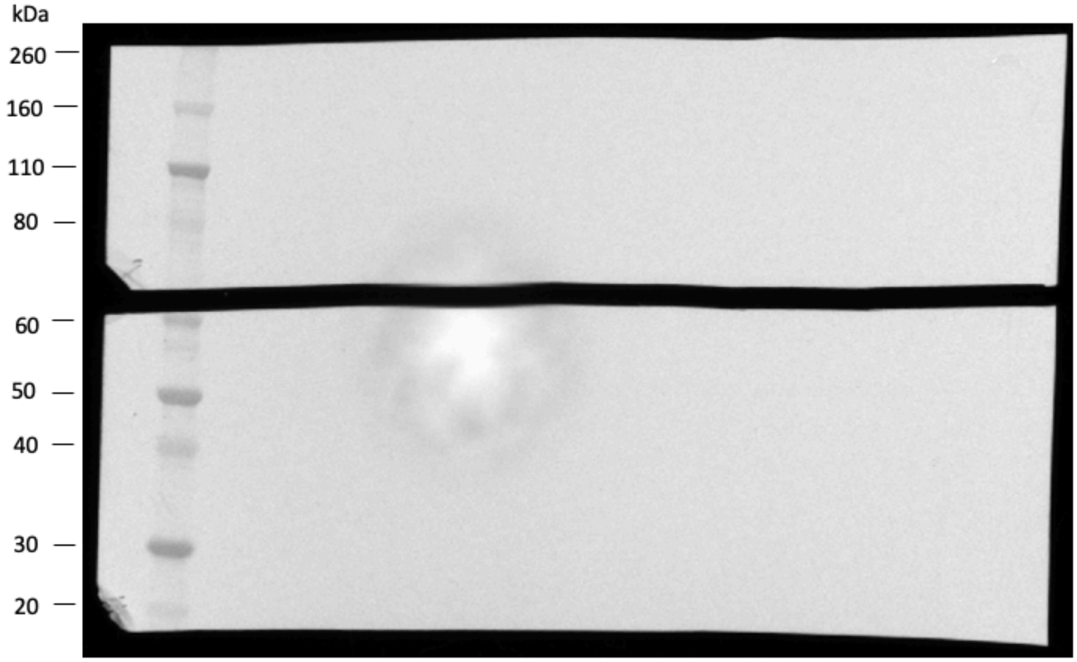

60-120 minutes:

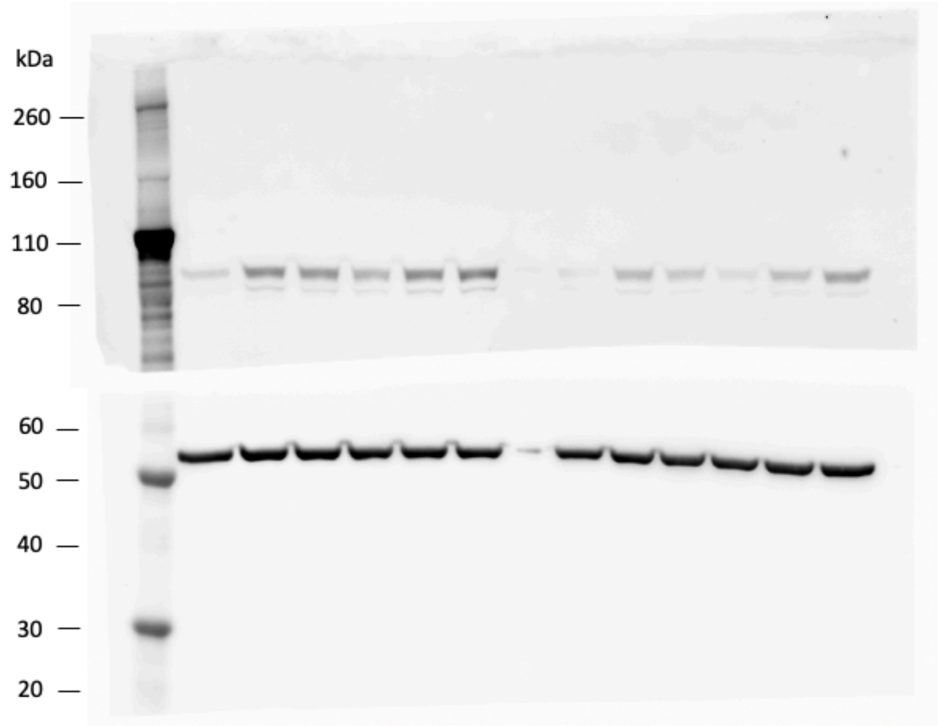

Colorimetric ladder image:

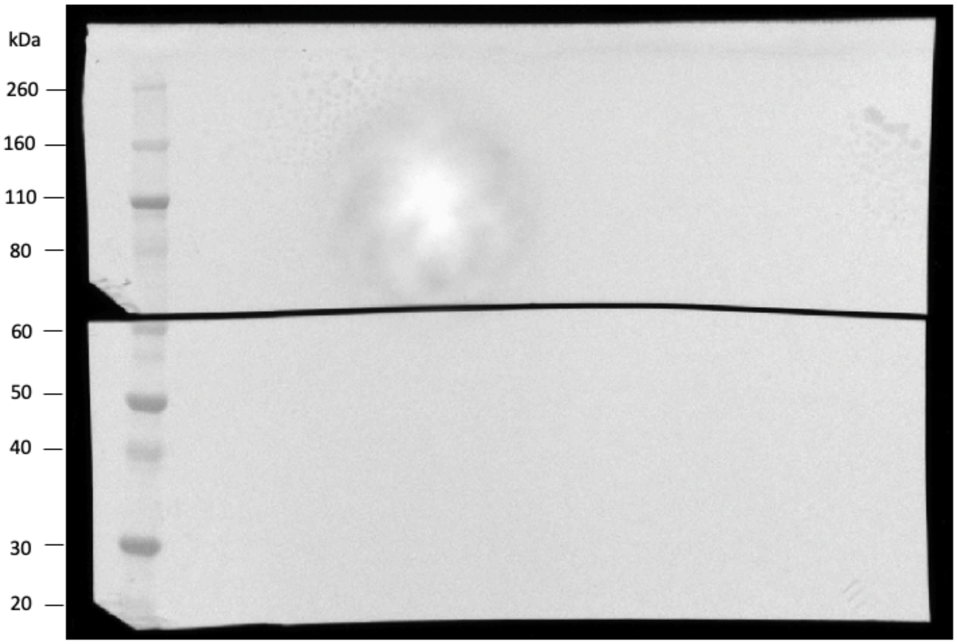

Supplementary Figure 4.

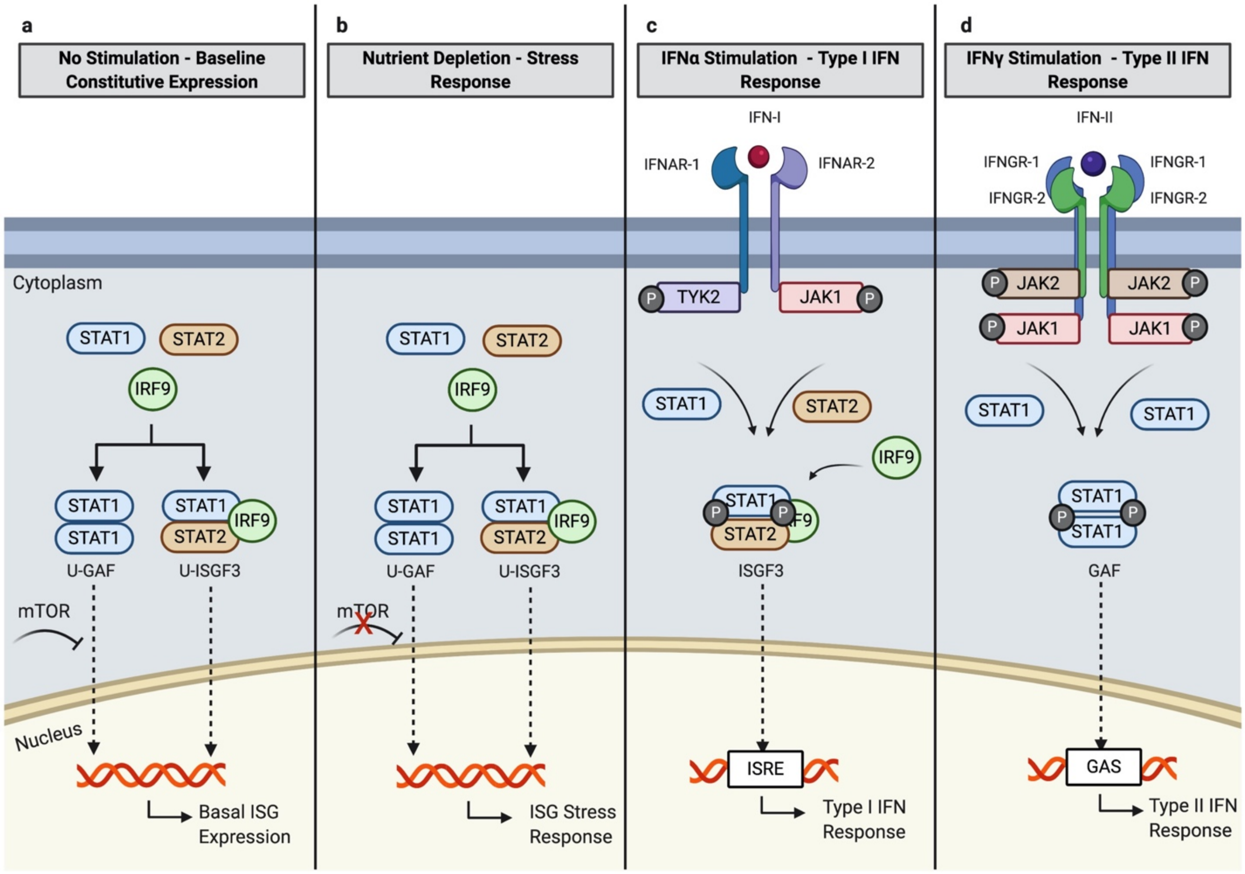

Supplementary Table 1: pSTAT1/Tubulin following IFNγ stimulation - densitometry analysis of immunoblots

Genotype	Experiment	pSTAT1/Tubulin	pSTAT1/Tubulin - percentage of WT per each experiment	Mean (% of WT)
WT	1	1.659691759	100	100
WT	2	0.685576707	100	
WT	3	0.19264795	100	
WT	4	0.446300028	100	
WT	5	0.572328025	100	
E235G	1	2.631382444	158.5464548	149.466837
E235G	2	0.919113296	134.0642537	
E235G	3	0.2994283	155.4277115	
E235G	4	0.67909645	152.1614178	
E235G	5	0.842091116	147.1343494	
K278E	1	3.472278943	209.2122784	177.869135
K278E	2	1.115974038	162.7788731	
K278E	3	0.358083084	185.8743288	
K278E	4	0.852208894	190.9497737	
K278E	5	0.804294992	140.5304226	
P329L	1	3.013079363	181.5445155	165.478582
P329L	2	0.829695249	121.021505	
P329L	3	0.391353254	203.1442608	
P329L	4	0.821830124	184.1429694	
P329L	5	0.78717802	137.5396601	
T385M	1	3.57198519	215.2197943	171.189318
T385M	2	0.931353687	135.849669	
T385M	3	0.328079882	170.3002196	
T385M	4	0.725562602	162.5728336	
T385M	5	0.984427519	172.0040739	
D517G	1	3.332493409	200.7898991	156.853892
D517G	2	1.129603874	164.7669564	
D517G	3	0.270072566	140.1896911	
D517G	4	0.509445939	114.1487579	
D517G	5	0.940759359	164.3741557	
Y701C	1	0.729638157	43.96226908	58.2630681
Y701C	2	0.402104115	58.65195111	
Y701C	3	0.139625945	72.47725449	
Y701C	4	0.270690276	60.65208575	

Supplementary Table 2: STAT1/Tubulin following IFNγ stimulation - densitometry analysis of immunoblots

Genotype	Experiment	STAT1/Tubulin	STAT1/Tubulin - percentage of WT per each experiment	Mean (% of WT)
WT	1	0.665257597	100	100
WT	2	0.206643538	100	
WT	3	0.036689368	100	
WT	4	0.091110442	100	
WT	5	0.153267657	100	
E235G	1	0.771844577	116.021911	120.332413
E235G	2	0.204002359	98.72186713	
E235G	3	0.054454786	148.4211637	
E235G	4	0.105887354	116.2186819	
E235G	5	0.1874133	122.2784398	
K278E	1	0.602361233	90.54556252	144.461651
K278E	2	0.267441414	129.4216196	
K278E	3	0.065428504	178.3309662	
K278E	4	0.133560361	146.591717	
K278E	5	0.271925006	177.4183879	
P329L	1	0.551294868	82.86938335	167.684037
P329L	2	0.208465538	100.8817113	
P329L	3	0.096005826	261.6720649	
P329L	4	0.187726066	206.0423176	
P329L	5	0.286541103	186.9547096	
T385M	1	1.108843999	166.6788932	177.111977
T385M	2	0.300709447	145.5208568	
T385M	3	0.079580199	216.902619	
T385M	4	0.15346647	168.440046	
T385M	5	0.288169973	188.0174714	
D517G	1	0.844711143	126.9750464	165.024779
D517G	2	0.247791279	119.9124258	
D517G	3	0.078875104	214.980821	
D517G	4	0.145815606	160.0426951	
D517G	5	0.311459657	203.2129045	
Y701C	1	0.493416669	74.16926491	109.682524
Y701C	2	0.11732849	56.77820418	
Y701C	3	0.05849544	159.4343093	
Y701C	4	0.118846143	130.4418473	
Y701C	5	0.195552665	127.5889961	

Supplementary Table 3: pSTAT1/Tubulin time-course (0-120 minutes) following IFNγ stimulation - densitometry analysis of immunoblots

Genotype	Time (minutes)	pSTAT1/Tubulin (Experiment 1)	pSTAT1/Tubulin (Experiment 2)
WT	0	0	0
E235G	0	0	0
K278E	0	0	0
P329L	0	0	0
T385M	0	0	0
D517G	0	0	0
WT	30	0.515511699	0.365437524
E235G	30	0.769238443	0.628864556
K278E	30	0.877176913	0.654588814
P329L	30	1.199901236	0.892381925
T385M	30	1.076605795	0.832291756
D517G	30	0.653600782	0.521782828
WT	60	0.664165873	0.408095512
E235G	60	1.152674563	0.686435723
K278E	60	1.047935362	0.77194422
P329L	60	0.843878989	0.719382132
T385M	60	1.111218772	1.019797628
D517G	60	1.031227634	0.879318339
WT	120	0.487592092	0.477744952
E235G	120	0.657126093	0.709691529
K278E	120	0.657937344	0.746896951
P329L	120	0.696400292	0.830138398
T385M	120	1.239233963	0.927537664
D517G	120	1.005181707	0.690580674

Supplementary Table 4: pSTAT1/ de-phosphorylation assay: pSTAT1/Tubulin following IFNγ stimulation at 0-120 minutes from Ruxolitinib administration - densitometry analysis of immunoblots

Genotype	Time from Ruxolitinib (minutes)	pSTAT1/Tubulin (Experiment 1)	pSTAT1/Tubulin (Experiment 2)
WT	0	0.51393922	0.482987702
E235G	0	0.852454001	0.739001553
K278E	0	1.122602321	0.880777682
P329L	0	0.964469579	0.813779524
T385M	0	1.051368059	0.658004216
D517G	0	1.135336917	0.70739242
WT	30	0.285646355	0.223482225
E235G	30	0.564083716	0.507679867
K278E	30	0.528430857	0.471385588
P329L	30	0.460668163	0.379541217
T385M	30	0.609815591	0.500929576
D517G	30	0.695440543	0.420864395
WT	60	0.10986184	0.151570576
E235G	60	0.337741246	0.482463297
K278E	60	0.330810703	0.47763585
P329L	60	0.218865505	0.319542389
T385M	60	0.359097532	0.487394595
D517G	60	0.449198663	0.622756871
WT	120	0.050638687	0.087074315
E235G	120	0.204347538	0.257604925
K278E	120	0.143554476	0.154179105
P329L	120	0.074117974	0.082189422
T385M	120	0.159107162	0.226439287
D517G	120	0.278682813	0.348708894

Supplementary Table 5 (A-F): STAT1 nuclear fluorescence in unstimulated cells

(A) WILDTYPE:

	Area	Mean	Integrated Density	Raw
Background 1	328.28	97.675	32064.776	985541
Background 2	400.118	100.038	40027.018	1230268
Background 3	236.368	94.083	22238.299	683515
Background 4	197.684	106.485	21050.308	647001
Background 5	261.03	90.124	23525.131	723067
**Mean background fluorescence**		**97.681**		

	Area	Mean	Integrated Density	Raw	Corrected fluorescence
Nucleus 1	97.606	601.083	58669.071	1803249	49134.81931
Nucleus 2	75.449	795.595	60026.928	1844984	52656.99423
Nucleus 3	102.909	672.407	69196.649	2126824	59144.39497
Nucleus 4	83.941	639.679	53695.219	1650373	45495.77818
Nucleus 5	98.614	741.804	73152.377	2248407	63519.66287
Nucleus 6	130.564	730.27	95346.786	2930573	82593.16392
Nucleus 7	132.548	797.332	105685.074	3248330	92737.65281
Nucleus 8	91.879	748.003	68726.06	2112360	59751.2274
Nucleus 9	96.207	729.955	70226.486	2158477	60828.89003
Nucleus 10	95.1	672.464	63951.617	1965613	54662.1539
**Mean corrected fluorescence**					**62052.47376**

(B) E235G:

	Area	Mean	Integrated Density	Raw
Background 1	189.387	86.041	16294.996	500842
Background 2	639.414	101.193	64704.091	1988741
Background 3	189.745	92.121	17479.505	537249
Background 4	426.179	78.146	33304.204	1023636
Background 5	563.868	99.268	55973.822	1720408
**Mean background fluorescence**		**91.3538**		

	Area	Mean	Integrated Density	Raw	Corrected fluorescence
Nucleus 1	135.574	531.659	72079.203	2215422	59694.00292
Nucleus 2	124.512	481.834	59994.165	1843977	48619.52065
Nucleus 3	106.358	555.576	59089.686	1816177	49373.47854
Nucleus 4	89.537	549.42	49193.356	1512004	41013.81081
Nucleus 5	97.736	523.395	51154.448	1572280	42225.893
Nucleus 6	107.073	473.59	50708.846	1558584	40927.32057
Nucleus 7	90.09	535.981	48286.535	1484132	40056.47116
Nucleus 8	106.293	527.172	56034.403	1722270	46324.13354
Nucleus 9	103.787	489.559	50810.03	1561694	41328.69316
Nucleus 10	127.505	574.368	73235.114	2250950	61587.04773
**Mean corrected fluorescence**					**47115.03721**

(C) K278E:

	Area	Mean	Integrated Density	Raw
Background 1	160.431	57.055	9153.324	281336
Background 2	115.24	77.621	8945.001	274933
Background 3	184.312	69.377	12787.083	393023
Background 4	172.014	59.1	10165.982	312461
Background 5	150.573	71.836	10816.556	332457
**Mean background fluorescence**		**66.9978**		

	Area	Mean	Integrated Density	Raw	Corrected fluorescence
Nucleus 1	88.138	818.366	72129.014	2216953	66223.9619
Nucleus 2	107.626	738.963	79531.944	2444489	72321.23878
Nucleus 3	82.151	594.059	48802.738	1499998	43298.80173
Nucleus 4	85.112	895.282	76199.363	2342059	70497.04625
Nucleus 5	120.575	940.762	113432.812	3486464	105354.5523
Nucleus 6	61.524	491.983	30268.768	930339	26146.79535
Nucleus 7	99.753	554.702	55333.107	1700715	48649.87546
Nucleus 8	73.367	843.867	61911.855	1902919	56996.42741
Nucleus 9	79.451	542.695	43117.602	1325260	37794.55979
Nucleus 10	99.07	664.006	65782.861	2021898	59145.38895
**Mean corrected fluorescence**					**58642.86479**

(D) P329L:

	Area	Mean	Integrated Density	Raw
Background 1	241.737	266.806	64496.874	1982372
Background 2	147.872	202.049	29877.532	918314
Background 3	143.155	240.452	34421.919	1057990
Background 4	116.671	226.139	26383.967	810936
Background 5	178.781	281.595	50343.768	1547363
**Mean background fluorescence**		**243.4082**		

	Area	Mean	Integrated Density	Raw	Corrected fluorescence
Nucleus 1	98.712	1323.731	130667.847	4016199	106640.5368
Nucleus 2	78.768	922.898	72694.541	2234335	53521.7639
Nucleus 3	70.894	1435.576	101774.05	3128121	84517.86907
Nucleus 4	94.124	1257.722	118382.229	3638589	95471.67558
Nucleus 5	96.727	1294.547	125217.843	3848688	101673.698
Nucleus 6	129.392	943.56	122089.616	3752539	90594.54219
Nucleus 7	87.097	1323.716	115291.32	3543587	94091.196
Nucleus 8	95.1	1326.248	126126.746	3876624	102978.6262
Nucleus 9	81.208	1417.978	115150.996	3539274	95384.30289
Nucleus 10	95.816	1289.811	123584.738	3798493	100262.3379
**Mean corrected fluorescence**					**92513.65485**

(E) T385M:

	Area	Mean	Integrated Density	Raw
Background 1	152.362	82.071	12504.547	384339
Background 2	126.562	86.109	10898.122	334964
Background 3	134.468	67.239	9041.533	277900
Background 4	154.152	86.394	13317.765	409334
Background 5	100.924	72.836	7350.939	225938
**Mean background fluorescence**		**78.9298**		

	Area	Mean	Integrated Density	Raw	Corrected fluorescence
Nucleus 1	119.274	940.767	112209.13	3448853	102794.857
Nucleus 2	90.903	759.855	69073.406	2123036	61898.45039
Nucleus 3	81.208	1008.184	81872.494	2516428	75462.7628
Nucleus 4	69.365	909.572	63092.492	1939207	57617.52642
Nucleus 5	75.286	1132.812	85285.404	2621327	79343.09508
Nucleus 6	96.402	1078.935	104011.235	3196883	96402.24442
Nucleus 7	71.285	1140.888	81327.79	2499686	75701.27921
Nucleus 8	82.477	1136.813	93760.597	2881820	87250.70389
Nucleus 9	107.529	951.545	102318.526	3144856	93831.28354
Nucleus 10	121.356	861.123	104502.745	3211990	94924.14019
**Mean corrected fluorescence**					**82522.6343**

(F) D517G:

	Area	Mean	Integrated Density	Raw
Background 1	345.036	28.344	9779.854	300593
Background 2	161.505	32.509	5250.336	161374
Background 3	376.172	25.517	9598.958	295033
Background 4	397.515	29.72	11814.313	363124
Background 5	258.004	24.879	6418.935	197292
**Mean background fluorescence**		**28.1938**		

	Area	Mean	Integrated Density	Raw	Corrected fluorescence
Nucleus 1	146.831	479.636	70425.634	2164598	66285.91015
Nucleus 2	103.82	564.998	58657.977	1802908	55730.89668
Nucleus 3	152.623	568.343	86741.94	2666095	82438.91766
Nucleus 4	101.119	345.439	34930.542	1073623	32079.61314
Nucleus 5	127.408	362.174	46143.832	1418274	42551.71633
Nucleus 6	116.118	491.908	57119.484	1755621	53845.67633
Nucleus 7	97.541	474.978	46329.64	1423985	43579.58855
Nucleus 8	94.482	548.903	51861.601	1594015	49197.79439
Nucleus 9	89.114	505.134	45014.437	1383561	42501.97471
Nucleus 10	127.408	550.085	70085.153	2154133	66493.03733
**Mean corrected fluorescence**					**53470.51253**

Supplementary Table 6 (A-F): STAT1 nuclear fluorescence in cells after 60 minutes of IFNγ stimulation:

(A) WILDTYPE:

	Area	Mean	Integrated Density	Raw
Background 1	122.072	67.949	8294.622	254943
Background 2	219.873	74.938	16476.803	506430
Background 3	147.677	73.422	10842.747	333262
Background 4	176.471	69.872	12330.451	378988
Background 5	85.145	84.651	7207.556	221531
**Mean background fluorescence**		**74.1664**		

	Area	Mean	Integrated Density	Raw	Corrected fluorescence
Nucleus 1	102.128	919.442	93900.824	2886130	86326.3579
Nucleus 2	111.758	829.193	92669.301	2848278	84380.61247
Nucleus 3	77.954	732.312	57086.917	1754620	51305.34945
Nucleus 4	109.546	875.059	95859.182	2946322	87734.54955
Nucleus 5	79.679	754.144	60089.2	1846898	54179.69541
Nucleus 6	92.855	909.266	84430.314	2595045	77543.59293
Nucleus 7	90.773	917.715	83303.913	2560424	76571.60637
Nucleus 8	88.723	999.174	88650.227	2724748	82069.96149
Nucleus 9	108.928	815.788	88862.032	2731258	80783.23438
Nucleus 10	82.444	1258.94	103792.273	3190153	97677.69832
**Mean corrected fluorescence**					**77857.26583**

(B) E235G:

	Area	Mean	Integrated Density	Raw
Background 1	82.347	127.565	10504.576	322868
Background 2	65.884	123.586	8142.325	250262
Background 3	40.962	121.589	4980.521	153081
Background 4	80.232	122.873	9858.329	303005
Background 5	92.628	120.275	11140.802	342423
**Mean background fluorescence**		**123.1776**		

	Area	Mean	Integrated Density	Raw	Corrected fluorescence
Nucleus 1	119.079	2274.955	270899.059	8326337	256231.1936
Nucleus 2	172.079	1319.258	227016.164	6977555	205819.8858
Nucleus 3	140.097	1424.532	199572.005	6134033	182315.1928
Nucleus 4	177.61	1453.177	258098.311	7932894	236220.7375
Nucleus 5	106.878	1779.634	190203.981	5846098	177039.0055
Nucleus 6	103.364	2008.876	207646.168	6382200	194914.0386
Nucleus 7	131.149	1791.95	235013.121	7223349	218858.5019
Nucleus 8	141.756	1428.319	202472.681	6223188	185011.5171
Nucleus 9	116.834	1622.67	189582.852	5827007	175191.5203
Nucleus 10	118.786	1556.875	184934.97	5684150	184934.97
**Mean corrected fluorescence**					**201653.6563**

(C) K278E:

	Area	Mean	Integrated Density	Raw
Background 1	92.855	98.617	9157.163	281454
Background 2	57.36	93.42	5358.515	164699
Background 3	94.58	93.384	8832.201	271466
Background 4	93.701	97.519	9137.707	280856
Background 5	144.717	94.227	13636.187	419121
**Mean background fluorescence**		**95.4334**		

	Area	Mean	Integrated Density	Raw	Corrected fluorescence
Nucleus 1	104.601	2420.746	253211.654	7782698	243229.2249
Nucleus 2	94.092	2740.624	257870.305	7925886	248890.7855
Nucleus 3	121.519	2269.623	275802.309	8477043	264205.3377
Nucleus 4	108.049	1746.185	188674.274	5799081	178362.7906
Nucleus 5	84.592	2505.983	211984.965	6515557	203912.0628
Nucleus 6	118.656	1615.942	191741.009	5893340	180417.2635
Nucleus 7	140.129	1772.247	248343.347	7633066	234970.3601
Nucleus 8	113.45	1742.892	197731.553	6077465	186904.6338
Nucleus 9	118.558	2615.325	310068.417	9530244	298754.024
Nucleus 10	122.528	2324.865	284860.044	8755441	273166.7804
**Mean corrected fluorescence**					**231281.3263**

(D) P329L:

	Area	Mean	Integrated Density	Raw
Background 1	110.359	126.21	13928.418	428103
Background 2	44.183	123.37	5450.818	167536
Background 3	62.825	138.685	8712.96	267801
Background 4	48.64	125.726	6115.349	187961
Background 5	69.658	124.713	8687.224	267010
**Mean background fluorescence**		**127.7408**		

	Area	Mean	Integrated Density	Raw	Corrected fluorescence
Nucleus 1	99.981	1118.401	111818.447	3436845	99046.79408
Nucleus 2	80.004	1545.71	123663.116	3800902	113443.341
Nucleus 3	87.552	965.445	84526.846	2598012	73342.88348
Nucleus 4	95.523	1098.764	104957.587	3225970	92755.40256
Nucleus 5	91.684	1216.145	111501.294	3427097	99789.50649
Nucleus 6	100.469	957.172	96165.859	2955748	83331.86856
Nucleus 7	95.133	1024.743	97486.821	2996349	85334.45547
Nucleus 8	105.479	1118.768	118006.708	3627047	104532.7362
Nucleus 9	75.221	1376.033	103507.102	3181388	93898.31128
Nucleus 10	82.249	1544.468	127030.899	3904414	116524.3459
**Mean corrected fluorescence**					**96199.96451**

(E) T385M:

	Area	Mean	Integrated Density	Raw
Background 1	89.504	74.581	6675.28	205171
Background 2	54.106	77.026	4167.564	128094
Background 3	121.031	64.992	7866.101	241772
Background 4	110.62	63.829	7060.79	217020
Background 5	110.652	76.443	8458.534	259981
**Mean background fluorescence**		**71.3742**		

	Area	Mean	Integrated Density	Raw	Corrected fluorescence
Nucleus 1	125.521	1669.304	209532.429	6440176	200573.468
Nucleus 2	103.104	1689.392	174183.127	5353682	166824.1615
Nucleus 3	119.079	1404.208	167211.451	5139401	158712.2826
Nucleus 4	86.381	1523.65	131614.394	4045292	125449.0192
Nucleus 5	87.813	1546.704	135819.927	4174553	129552.3444
Nucleus 6	124.805	1696.648	211750.159	6508340	202842.302
Nucleus 7	88.17	1531.635	135044.873	4150731	128751.8098
Nucleus 8	79.776	1546.96	123410.74	3793145	117716.7918
Nucleus 9	77.629	1460.112	113347.049	3483828	107806.3412
Nucleus 10	106.423	1197.341	127424.25	3916504	119828.3935
**Mean corrected fluorescence**					**145805.6914**

(F) D517G:

	Area	Mean	Integrated Density	Raw
Background 1	109.481	39.995	4378.685	134583
Background 2	103.397	42.029	4345.662	133568
Background 3	182.23	39.615	7219.008	221883
Background 4	102.128	39.176	4000.984	122974
Background 5	109.091	36.619	3994.835	122785
**Mean background fluorescence**		**39.4868**		

	Area	Mean	Integrated Density	Raw	Corrected fluorescence
Nucleus 1	110.717	1266.694	140244.974	4310561	135873.114
Nucleus 2	64.159	1238.4	79454.998	2442124	76921.5644
Nucleus 3	71.382	1135.147	81029.344	2490513	78210.69724
Nucleus 4	106.911	1291.526	138077.87	4243953	133856.2967
Nucleus 5	76.197	1087.705	82880.37	2547406	79871.5943
Nucleus 6	68.91	1119.283	77129.317	2370642	74408.28161
Nucleus 7	81.013	876.622	71017.514	2182790	67818.56987
Nucleus 8	93.864	1023.506	96070.433	2952815	92364.044
Nucleus 9	69.007	1106.927	76385.92	2347793	73661.05439
Nucleus 10	81.859	1132.661	92717.974	2849774	89485.62404
**Mean corrected fluorescence**					**90247.08406**
