# Supplementary material for: STAT1 gain-of-function heterozygous cell models reveal diverse interferon-signature gene transcriptional responses
Source: NPJ Genom Med. 2021 May 14;6:34. doi: 10.1038/s41525-021-00196-7 (PMC8121859; doi:10.1038/s41525-021-00196-7)
Supplement: Supplementary file 2 — Reporting Summary [file 41525_2021_196_MOESM2_ESM.pdf]

nature research

Corresponding author(s): Evgueni A. Ivakine
Ori Scott

Last updated by author(s): Nov 4, 2020

Reporting Summary

Nature Research wishes to improve the reproducibility of the work that we publish. This form provides structure for consistency and transparency in reporting. For further information on Nature Research policies, see our Editorial Policies and the Editorial Policy Checklist.

Statistics

For all statistical analyses, confirm that the following items are present in the figure legend, table legend, main text, or Methods section.

n/a	Confirmed	
☐	☒	The exact sample size (*n*) for each experimental group/condition, given as a discrete number and unit of measurement
☐	☒	A statement on whether measurements were taken from distinct samples or whether the same sample was measured repeatedly
☐	☒	The statistical test(s) used AND whether they are one- or two-sided<br>*Only common tests should be described solely by name; describe more complex techniques in the Methods section.*
☐	☒	A description of all covariates tested
☐	☒	A description of any assumptions or corrections, such as tests of normality and adjustment for multiple comparisons
☐	☒	A full description of the statistical parameters including central tendency (e.g. means) or other basic estimates (e.g. regression coefficient) AND variation (e.g. standard deviation) or associated estimates of uncertainty (e.g. confidence intervals)
☐	☒	For null hypothesis testing, the test statistic (e.g. *F*, *t*, *r*) with confidence intervals, effect sizes, degrees of freedom and *P* value noted<br>*Give P values as exact values whenever suitable.*
☒	☐	For Bayesian analysis, information on the choice of priors and Markov chain Monte Carlo settings
☒	☐	For hierarchical and complex designs, identification of the appropriate level for tests and full reporting of outcomes
☒	☐	Estimates of effect sizes (e.g. Cohen's *d*, Pearson's *r*), indicating how they were calculated

Our web collection on statistics for biologists contains articles on many of the points above.

Software and code

Policy information about availability of computer code

Data collection	DNA sequencing data was obtained using Applied Biosystems SeqStudio Genetic Analyzer.<br>Quantitative real-time PCR data was collected using Applied Biosystems QuantStudio 3 Real-Time PCR System.<br>Immunoblot data was collected using ChemiDoc MP imaging system (Bio-Rad).<br>Flow cytometry data was collected using BD LSR II and the BD FACSDiva software
Data analysis	Analysis of bulk population base-editing frequency was performed using the free online software EditR version 1.0.9 available at: https://moriaritylab.shinyapps.io/editr_v10/<br>Immunoblot images were analyzed via Image Lab software (©2017 Bio-Rad Laboratories; version 6.0.1).<br>Data graphing and statistical analysis has been done using GraphPad Prism 8 (version 8.4.3).<br>Flow cytometry data was analyzed using FlowJo 10.7.1

For manuscripts utilizing custom algorithms or software that are central to the research but not yet described in published literature, software must be made available to editors and reviewers. We strongly encourage code deposition in a community repository (e.g. GitHub). See the Nature Research guidelines for submitting code & software for further information.

Data

Policy information about availability of data

All manuscripts must include a data availability statement. This statement should provide the following information, where applicable:

- Accession codes, unique identifiers, or web links for publicly available datasets
- A list of figures that have associated raw data
- A description of any restrictions on data availability

The datasets generated during and/or analysed during the current study are available from the corresponding author on reasonable request

Field-specific reporting

Please select the one below that is the best fit for your research. If you are not sure, read the appropriate sections before making your selection.

☒ Life sciences ☐ Behavioural & social sciences ☐ Ecological, evolutionary & environmental sciences

For a reference copy of the document with all sections, see nature.com/documents/nr-reporting-summary-flat.pdf

Life sciences study design

All studies must disclose on these points even when the disclosure is negative.

Sample size	Protein studies: Studies examining phospho-STAT1 and total STAT1 levels across the various genotypes before and after interferon stimulation were performed independently a total of 5 times. Samples from independent experiments were subjected to protein electrophoresis separately, and analyzed separately by densitometry. Studies examining the phosphorylation and/or de-phosphorylation kinetics of pSTAT1 were performed in duplicates, subjected to protein electrophoresis separately, and analyzed separately by densitometry.<br><br>RNA gene expression studies: studies examining gene expression across the various genotypes were each performed a minimum of 5 times, and each sample was run via at least technical duplicates.<br>The sample sizes chosen above are in keeping with, or above, what is commonly published in similar studies
Data exclusions	For gene expression studies, samples for which a Ct value could not be established were followed by repeated measurement compared with the appropriate controls. Following this measure, no data were excluded from the analysis
Replication	Data replicability was ensured by inclusion of data from independent experiments. All data included in this study has been replicable in our lab
Randomization	Not applicable
Blinding	Blinding during data analysis in this study was not possible, as the individual performing the data analysis (OS) also performed considerable parts of the data collection. We believe blinding not to be applicable for this type of study as no therapeutic intervention was tested and no human subjects involved

Reporting for specific materials, systems and methods

We require information from authors about some types of materials, experimental systems and methods used in many studies. Here, indicate whether each material, system or method listed is relevant to your study. If you are not sure if a list item applies to your research, read the appropriate section before selecting a response.

Materials & experimental systems

n/a	Involved in the study	
☐	☒	Antibodies
☐	☒	Eukaryotic cell lines
☒	☐	Palaeontology and archaeology
☒	☐	Animals and other organisms
☒	☐	Human research participants
☒	☐	Clinical data
☒	☐	Dual use research of concern

Methods

n/a	Involved in the study	
☒	☐	ChIP-seq
☐	☒	Flow cytometry
☒	☐	MRI-based neuroimaging

Antibodies

Antibodies used	The following primary antibodies were used for the purpose of immunoblotting and/or immunofluorescence:<br>Rabbit anti-pSTAT1 (pY701; clone D4A7; Cell Signaling 7649S)<br>Rabbit anti-Total STAT1 (Cell Signaling; clone D1K9Y; 14994)<br>Mouse anti-alpha-tubulin (clone DM1A; Sigma Aldrich T6199-100UL).<br><br>The following secondary antibodies were used:<br>Donkey anti-Rabbit IgG (H+L) Highly Cross-Adsorbed Secondary Antibody, Alexa Fluor 647 (Thermo Fisher Scientific A-31573)<br>Donkey anti-Mouse IgG (H+L) Highly Cross-Adsorbed Secondary Antibody, Alexa Fluor 647 (Thermo Fisher Scientific A-31571).<br>Goat anti-Rabbit IgG (H+L) Cross-Adsorbed Secondary Antibody, Alexa Fluor 555 (Thermo Fisher Scientific A-21428).
Validation	1) Rabbit anti-pSTAT1 (pY701; clone D4A7; Cell Signaling 7649S): this antibody has been validated by the manufacturer to be reactive against human pSTAT1 (Tyr701). Western blotting proposed antibody concentration and sample images provided by the manufacturer website: https://www.cellsignal.com/products/primary-antibodies/phospho-stat1-tyr701-d4a7-rabbit-mab/7649. The antibody has been successfully validated by a number of studies. The website provides 12 citations which have used this antibody for Western Blotting, with Homo sapiens reactivity

2) Rabbit anti-Total STAT1 (Cell Signaling; clone D1K9Y; 14994): this antibody has been validated by the manufacturer to be reactive against human STAT1. Western blotting proposed antibody concentration and sample images provided by the manufacturer website: https://www.cellsignal.com/products/primary-antibodies/stat1-d1k9y-rabbit-mab/14994. The antibody has been successfully validated by a number of studies. The website provides 7 citations which have used this antibody for Western Blotting, with Homo sapiens reactivity

3) Mouse anti-alpha-tubulin (Clone DM1A; Sigma Aldrich T6199-100UL): this antibody has been validated by the manufacturer to be reactive against human Alpha-tubulin. Western blotting proposed antibody concentration and sample images provided by the manufacturer website: https://www.sigmaaldrich.com/catalog/product/sigma/t6199?lang=en&region=CA. The antibody has been successfully validated by a number of studies. The website provides a total of 1582 citations which have used this antibody, of which at least 10 used it for Western blotting with homo sapiens reactivity

Eukaryotic cell lines

Policy information about cell lines

Cell line source(s)	Wildtype HAP1 cells were kind gift of Dr. Aleixo Muise lab (Toronto). Wildtype human fibroblasts were purchased from ATCC
Authentication	Cell line authentication for HAP1 cells was done via short-tandem repeat (STR) analysis, performed by The Hopsital for Sick Children The Centre for Applied Genomics (TCAG)
Mycoplasma contamination	Cells were confirmed to be Mycoplasma-free using a PCR Mycoplasma Detection Kit (Applied Biological Materials Inc. G238).
Commonly misidentified lines (See ICLAC register)	N/A

Flow Cytometry

Plots

Confirm that:

☒ The axis labels state the marker and fluorochrome used (e.g. CD4-FITC).

☒ The axis scales are clearly visible. Include numbers along axes only for bottom left plot of group (a 'group' is an analysis of identical markers).

☒ All plots are contour plots with outliers or pseudocolor plots.

☒ A numerical value for number of cells or percentage (with statistics) is provided.

Methodology

Sample preparation	Flow cytometry was employed for the purpose of DNA-content analysis. HAP1 cells were compared with wildtype human fibroblasts known to be diploid. cells were washed with 1xPBS (Wisent Bioproducts 311-010-CL), trypsinized, and resuspended in complete media (1x106 cells/µl), to which a low-toxicity, cell-permeable DNA dye (Vybrant DyeCycle VioletTM; 1:1000, Thermo Fisher Scientific V35003) was added. Cells were incubated at 37°C for 30 minutes. Live/dead cell stain was concurrently performed using propidium iodide (1µg/µL; Thermo Fisher Scientific P1304MP), added according to manufacturer recommendation.
Instrument	Samples were run on BD LSR-II
Software	BD FACSDivaTM software v9.0 was used for data acquisition.<br>FlowJo 10.7.1 was used for data analysis
Cell population abundance	Following exclusion of cell debris, 64.8-70.8% were included for further analysis. Following single-cell discrimination by FSC, 68.2-93% of above cells were included for further analysis. Following second step of single-cell discrimination by SSC, 93-95.8% of above cells were included for further analysis. Following dead cell exclusion, 86.7-99.6% of above cells were included in the final analysis
Gating strategy	As a first step, plotting of FSC-A vs. SSC-A was used to gate out debris. Doublet exclusion subsequently followed using two steps, first using the FSC (FSC-A vs. FSC-H) followed by SSC doublet discrimination (SSC-A vs SSC-H). Then, dead cells were excluded by plotting the G660-A (propidium iodide) fluorescence intensity vs. SSC-H. Events associated with G660-A fluorescence intensity higher than 10^3 were excluded.

☒ Tick this box to confirm that a figure exemplifying the gating strategy is provided in the Supplementary Information.
